# Comprehensive Analysis of RUNX and TGF-β Mediated Regulation of Immune Cell Infiltration in Breast Cancer

**DOI:** 10.3389/fcell.2021.730380

**Published:** 2021-08-18

**Authors:** Liang Gao, Fangfang Zhou

**Affiliations:** Institutes of Biology and Medical Sciences, Soochow University, Suzhou, China

**Keywords:** RUNX, transcription factor, breast cancer, methylation, immune cell infiltration, TGF-β

## Abstract

Runt-related transcription factors (RUNXs) can serve as both transcription activators and repressors during biological development, including immune cell maturation. RUNX factors have both tumor-promoting and tumor-suppressive roles in carcinogenesis. Immune cell infiltration and the tumor immune microenvironment have been found to be key regulators in breast cancer progression, treatment response, and patient outcome. However, the relationship between the RUNX family and immune cell infiltration in breast cancer remains unclear. We performed a comprehensive analysis to reveal the role of RUNX factors in breast cancer. Analysis of patient data in the Oncomine database showed that the transcriptional levels of RUNX proteins in breast cancer were elevated. Kaplan–Meier plotter (KM plotter) analysis showed that breast cancer patients with higher expression of RUNX proteins had better survival outcomes. Through analysis of the UALCAN database, we found that the transcriptional levels of RUNX factors were significantly correlated with some breast cancer patient characteristics. cBio Cancer Genomics Portal (cBioPortal) analysis showed the proportions of different RUNX genomic alterations in various subclasses of breast cancer. We also performed gene ontology (GO) and pathway analyses for the significantly differentially expressed genes that were correlated with RUNX factors in breast cancer. TIMER database analysis showed that immune cell infiltration in breast cancer could be affected by the transcriptional level, mutation, and gene copy number of RUNX proteins. Using the Gene Set Cancer Analysis (GSCA) database, we analyzed the effects of RUNX gene methylation on the level of immune cell infiltration in breast cancer. We found that the methylation level changes of RUNX2 and RUNX3 had opposite effects on immune cell infiltration in breast cancer. We also analyzed the relationship between the methylation level of RUNX genes and the TGF-β signaling pathway using the TISIDB database. The results showed that the methylation levels of RUNX1 and RUNX3 were correlated with the expression of TGF-β1. In summary, our analysis found that the RUNX family members can influence the infiltration of various immune cells in breast cancer depending on their expression level, mutation, gene copy number, and methylation. The RUNX family is an important regulator of immune cell infiltration in breast cancer and may serve as a potential prognostic biomarker.

## Introduction

Breast cancer is the most common cancer type among women and is one of the leading causes of cancer-related deaths worldwide (Siegel et al., 2021). With improvements in diagnostic techniques and precise treatments, the overall prognosis of breast cancer patients has substantially improved in recent years. However, not all patients respond favorably to current therapy, and relapse is common. This is due to the complex nature of breast cancer pathogenesis, development, and metastasis. Currently, the role of tumor-infiltrating immune cells in breast cancers and their effects on tumor progression and immunotherapy are gaining attention (Azizi et al., 2018). Clinical data-based analysis has indicated a significant impact of tumor-infiltrating immune cells on the clinical outcome of breast cancer patients, including treatment response, recurrence, and death (Adams et al., 2014; Ali et al., 2014; Loi et al., 2014). However, the regulatory mechanisms of immune cell infiltration in breast cancer are not fully understood. It is necessary to characterize the immune microenvironment of breast cancer, develop biomarkers to facilitate precise patient stratification, and provide potential therapeutic molecular targets to modulate the breast cancer microenvironment.

The Runt-related transcription factor (RUNX) family consists of three members in mammals: RUNX1, RUNX2, and RUNX3. All three types of RUNX factors are expressed in the mammary gland, and play a regulatory role in physiological and pathological states. RUNX1 is expressed in all subpopulations of murine mammary epithelial cells, with the exception of secretory alveolar luminal cells (Van Bragt et al., 2014). RUNX2 is expressed not only in embryonic mammary glands but also in adult luminal and basal cell lineages (Owens et al., 2014). With RUNX3, inactivation and protein mislocalization occur during the early stages of breast cancer progression (Subramaniam et al., 2009). The RUNX family is of critical importance in developmental processes and tumorigenesis (Blyth et al., 2005). RUNX factors serve as activators or repressors during developmental processes (Shin et al., 2021). Likewise, the role of RUNX factors in cancer biology has also been found to be two-sided. The RUNX family has been reported to exert both tumor-suppressive and tumor-promoting roles in breast cancer. For example, RUNX1 has been shown to regulate the estrogen receptor-positive luminal lineage, and RUNX1 mutations may present as an additional genetic predisposition in breast cancer development (Van Bragt et al., 2014). RUNX2 has been shown to have an antagonistic role in estradiol-induced breast cancer proliferation (Chimge et al., 2012). RUNX3 destabilizes estrogen receptor alpha and suppresses its transcriptional activation, thus supporting its tumor-suppressive role in breast cancer (Huang B. et al., 2012). In contrast, RUNX2 has also been demonstrated to promote tumorigenesis in breast cancer. RUNX2 deletion prolongs overall survival (OS) in mice with breast cancer (Owens et al., 2014). Moreover, RUNX2 contributes to the bone metastasis of breast cancer in an integrin-dependent pathway (Li X.Q. et al., 2016).

Given the complex roles of RUNX family members in breast cancer, a comprehensive analysis is needed to reveal the relationship between these transcription factors and breast cancer. In this study, we analyzed the transcriptional changes in RUNX family members, promoter methylation level, RUNX gene alteration, and their relationship to immune cell infiltration as well as breast cancer patient prognosis. Our study indicates the important role of RUNX family members in breast cancer pathophysiology and may provide potential biomarkers or therapeutic targets to facilitate early breast cancer diagnosis and treatment.

## Materials and Methods

### Oncomine

The Oncomine database was used to compare the expression levels of the three RUNX members across a variety of cancer types. The *p*-value was set as 1E−4, and the fold change was set as 2.

### Gene Expression Profiling Interactive Analysis

The RUNX expression profile was analyzed using the Gene Expression Profiling Interactive Analysis (GEPIA) tool, which was developed based on The Cancer Genome Atlas (TCGA) and Genotype-Tissue Expression (GTEx) databases (Tang et al., 2017). The RUNX expression profiles were compared between different breast cancer stages.

### UALCAN

The UALCAN portal (Chandrashekar et al., 2017) was used to analyze how the clinical characteristics of breast cancer patients were related to RUNX expression profiles and RUNX promoter methylation status. The pan-cancer analysis of RUNX factor expression was also carried out using the UALCAN web.

### Kaplan–Meier Plotter

The relationship between RUNX expression levels and prognosis of breast cancer patients, was analyzed using the Kaplan–Meier plotter (KM plotter; Gyorffy et al., 2010). A total of 2,032 patients were analyzed, and they were split according to the median expression level of RUNX transcription factors. The two groups were compared to determine OS, distant metastasis-free survival (DMFS), relapse-free survival (RFS), and progression-free survival (PFS).

### cBio Cancer Genomics Portal

The alterations of RUNX members in breast cancer subtypes were analyzed using the Breast Invasive Carcinoma (TCGA, PanCancer Atlas) dataset, which includes information from 1,084 samples. The alteration types include mutation, fusion, amplification, deep deletion, and multiple alterations.

### LinkedOmics Analysis

LinkedOmics is a multi-omics database containing information on 32 cancer types from TCGA (Vasaikar et al., 2018). The significantly differentially expressed genes that were correlated with RUNX members were analyzed using the TCGA_BRCA cancer cohort (HiSeq RNA platform) in LinkedOmics. Kyoto Encyclopedia Genes and Genomes (KEGG) pathway analysis was performed using gene set enrichment analysis (GSEA). The genes were also classified using gene ontology (GO) according to: biological processes, cellular components, and molecular functions.

### TIMER Analysis

The TIMER web server (Li B. et al., 2016; Li et al., 2017) was used to analyze the infiltration of six types of immune cells in breast cancer, including B cells, CD4^+^ T cells, CD8^+^ T cells, neutrophils, macrophages, and dendritic cells. The gene module was used to evaluate the relationship between target gene expression and immune cell infiltration, whereas the mutation module was used to analyze the gene mutation with an abundance of immune infiltrates. The SCNA module was used to analyze the correlation between RUNX gene somatic copy number alterations and tumor infiltration levels.

### Gene Set Cancer Analysis

The Gene Set Cancer Analysis (GSCA) database (Liu C.J. et al., 2018) was used to analyze the relationship between RUNX factor methylation levels and the infiltration of six immune cell types: B cells, CD8^+^ T cells, CD4^+^ T cells, macrophages, neutrophils, and dendritic cells in breast cancer.

### TISIDB Analysis

TISIDB is an integrated web portal for the analysis of tumor-immune system interactions (Ru et al., 2019). The correlations between RUNX gene methylation and the expression of TGF-β1 and TGFBR1, two of the critical immunomodulators in breast cancer, were analyzed using TISIDB.

## Results

### Pan-Cancer Analysis of RUNX Family Member Expression

**FIGURE 1:**
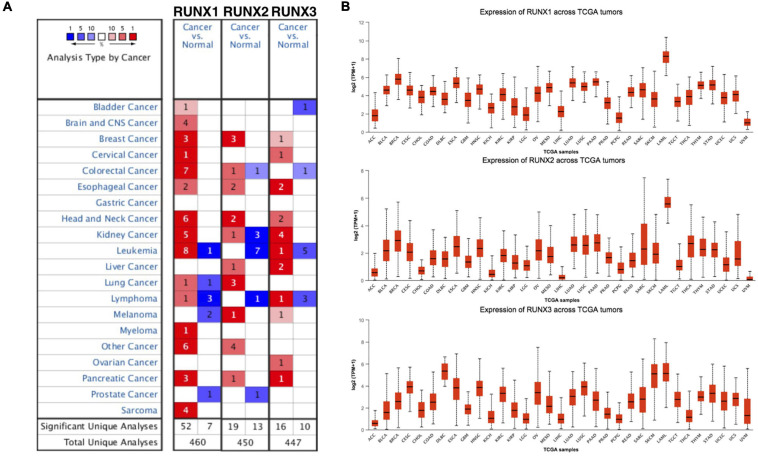
The transcriptional levels of Runt-related transcription factors (RUNXs) in different types of cancers. **(A)** The transcriptional levels of RUNX1, RUNX2, and RUNX3 in various cancer tissues compared with normal tissues. **(B)** The transcriptional levels of RUNX1, RUNX2, and RUNX3 were compared across various The Cancer Genome Atlas (TCGA) tumors.

To study the role of RUNX family members in cancer, we performed a pan-cancer analysis using Oncomine. Transcription levels of RUNX1, RUNX2, and RUNX3 were all increased in most types of cancers, including breast, esophageal, head and neck, and pancreatic cancer (Figure 1A). Expression levels of RUNX factors were then compared across TCGA tumors. RUNX1 and RUNX2 expression in breast cancer was relatively high, ranking second after acute myeloid leukemia (LAML) for all the analyzed tumor types, indicating their potential role in breast cancer (Figure 1B).

### Transcriptional Levels of RUNX Factors in Breast Cancer

**FIGURE 2:**
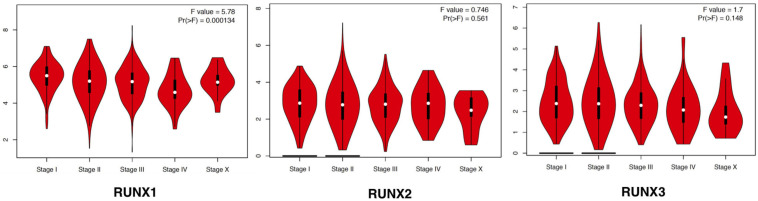
Correlation between RUNX expression and tumor stage in breast cancer patients. The transcriptional levels of RUNX1, RUNX2, and RUNX3 in different stages of breast cancer were shown.

**FIGURE 3:**
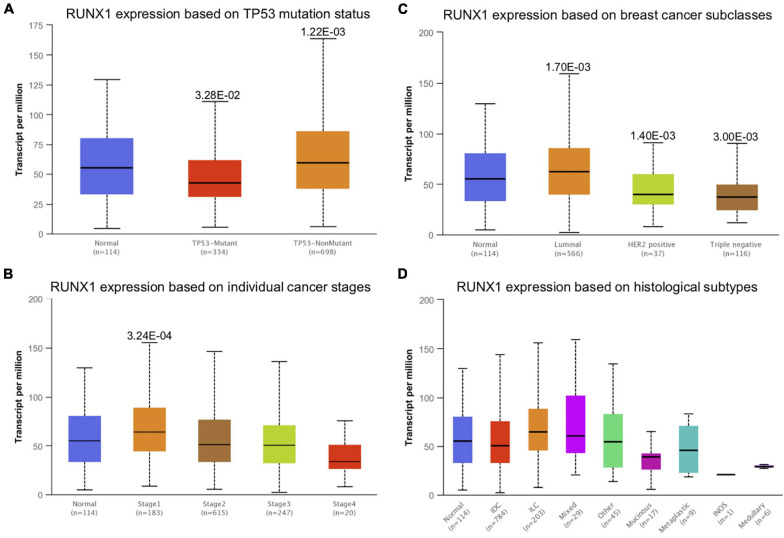
Correlation analysis between RUNX1 expression and breast cancer patient characteristics. The RUNX1 expression levels in breast cancer patients classified by **(A)** TP53 mutation status, **(B)** individual cancer stages, **(C)** breast cancer subclasses, and **(D)** histological subtypes were shown.

The expression of RUNX members in breast cancer cells was analyzed using GEPIA. We found that the expression level of RUNX1 was significantly associated with the stage of breast cancer (*p* = 0.0001), while there was no significant difference in RUNX2 and RUNX3 expression (Figure 2). To further dissect the relationship between RUNX expression levels and breast cancer patient characteristics, we performed UALCAN analysis. The transcriptional level of RUNX1 in breast cancer was found to be associated with the TP53 mutation status. Breast cancer patients with mutant TP53 had significantly lower RUNX1 levels than normal controls, whereas patients with non-mutant TP53 had significantly higher RUNX1 levels (Figure 3A). There was a decreasing trend in RUNX1 transcriptional levels from stage one to stage four in breast cancer patients (Figure 3B). There was also a significant difference in RUNX1 levels between different breast cancer subclasses, with luminal type having the highest level and triple-negative showing the lowest level (Figure 3C). Furthermore, RUNX1 transcriptional levels were associated with different histological types (Figure 3D). Similarly, we also used the UALCAN database to further analyze the expression levels of RUNX2 and RUNX3 in breast cancer. We found that RUNX2 expression significantly varied in different stages, subclasses, and histological subtypes of breast cancer (Supplementary Figure 1). RUNX3 was down-regulated in luminal subclass but up-regulated in triple-negative subclass (Supplementary Figure 2). To validate these findings, we compared the levels of RUNX transcripts in different breast cancer subtypes to those of normal controls (Table 1). RUNX transcriptional levels have been shown to be significantly increased in various subtypes of breast cancer.

**TABLE 1:** The significant changes of Runt-related transcription factor (RUNX) transcriptional levels in different types of breast cancers vs. normal breast tissues (oncomine database).

	Type of breast cancer vs. normal breast tissue	Fold change	*p*-Value	*t* Test	Source and references
RUNX1	Invasive lobular breast carcinoma vs. normal	2.002	1.21E−11	7.745	TCGA
	Invasive breast carcinoma vs. normal	2.102	2.03E−17	9.804	TCGA
	Fibroadenoma vs. normal	2.494	0.016	4.036	Sorlie Breast 2 Statistics
	Fibroadenoma vs. normal	2.271	0.033	2.886	Sorlie Breast Statistics
	Invasive ductal breast carcinoma stroma vs. Normal	2.124	0.01	2.587	Karnoub Breast Statistics
RUNX2	Invasive breast carcinoma vs. normal	2.307	9.11E−14	8.192	TCGA
	Invasive lobular breast carcinoma vs. normal	2.053	3.62E−09	6.477	TCGA
	Invasive ductal breast carcinoma stroma vs. normal	4.617	1.54E−04	4.523	Karnoub Breast Statistics
	Invasive ductal breast carcinoma vs. normal	2.932	0.047	2.02	Radvanyi Breast Statistics
	Invasive mixed breast carcinoma vs. normal	3.08	0.044	2.014	Radvanyi Breast Statistics
RUNX3	Medullary breast carcinoma vs. normal	2.321	9.17E−08	6.396	Curtis Breast Statistics

*TCGA, The Cancer Genome Atlas.*

### Survival Analysis

**FIGURE 4:**
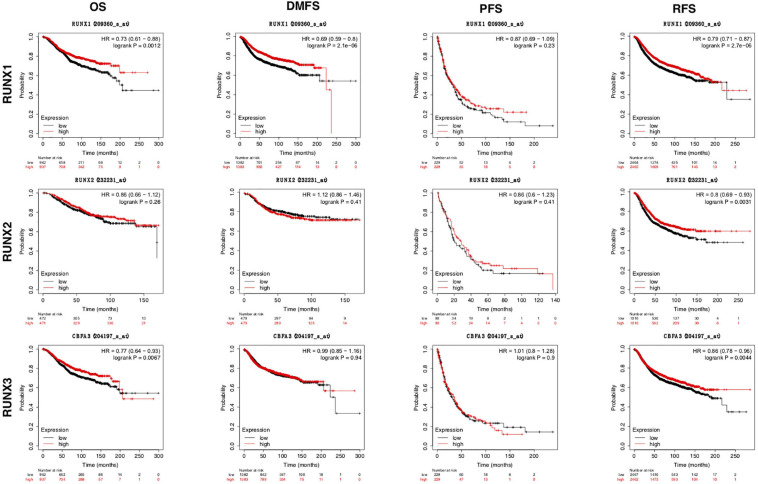
The prognostic value of RUNX transcriptional level in breast cancer patients. The breast cancer patients were classified into two groups by the median expression levels of RUNX1, RUNX2, or RUNX3. The overall survival (OS), distant metastasis-free survival (DMFS), progression-free survival (PFS), and relapse-free survival (RFS) were compared between patients with high or low expression of RUNX transcription factors.

To study the relationship between RUNX transcription factor expression levels and the outcomes of patients with breast cancer, we performed KM plotter analysis. The results showed that patients with higher RUNX levels had longer DMFS, OS, and RFS (Figure 4). Specifically, higher RUNX1 expression was associated with longer DMFS, OS, and RFS. Higher RUNX2 expression was associated with longer RFS. Higher RUNX3 expression was associated with longer OS and RFS. These results indicate that RUNX1 and RUNX3 may play a more important role in the OS and RFS of breast cancer patients, and RUNX2 only affects the RFS of breast cancer patients.

### Analysis of RUNX Alterations in Breast Cancer

**FIGURE 5:**
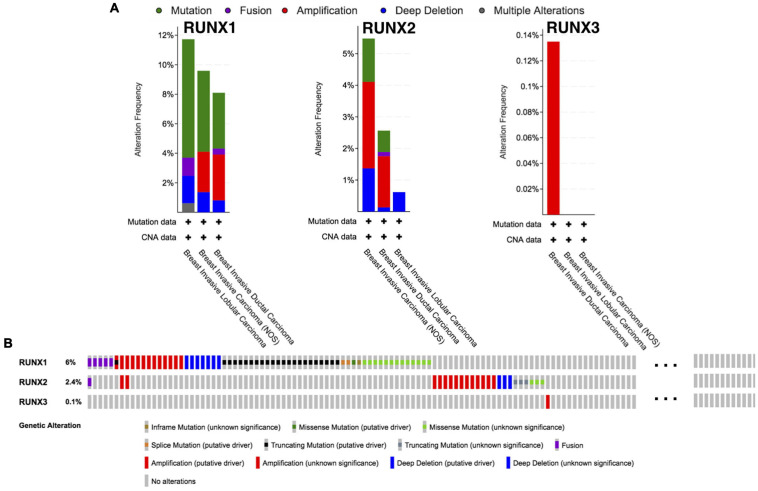
Runt-related transcription factor alternation analysis in breast cancer. **(A)** The proportions of various RUNX alternations in different subclasses of breast cancer. **(B)** The genetic alternations of RUNX1, RUNX2, and RUNX3 in breast cancer patients.

The alterations in RUNX members in breast cancer subtypes were analyzed using cBio Cancer Genomics Portal (cBioPortal). Alteration types include mutation, fusion, amplification, deep deletion, and multiple alterations. Interestingly, the three RUNX members seemed to be altered in different subtypes of breast cancer with varying tendencies. The subtypes with the highest frequency of all alternate types were breast invasive lobular carcinoma, breast invasive carcinoma NOS, and breast invasive ductal carcinoma for RUNX1, RUNX2, and RUNX3, respectively (Figure 5A). For RUNX1, mutation ranked first in all alternate types in breast invasive lobular carcinoma (8.02%), followed by breast invasive carcinoma NOS (4.11%), and breast invasive ductal carcinoma (3.10%). RUNX1 was mainly distributed in the Runt domain and the linker region (Supplementary Figure 3). For RUNX2, the alteration with the highest frequency was amplification, with a percentage of 2.74% in breast invasive carcinoma NOS and 1.62% in breast invasive ductal carcinoma. For RUNX3, alterations were mainly found to be amplified in breast invasive ductal carcinoma (0.13%). RUNX1 showed the highest number of alterations among the three members (Figure 5B).

### Analysis of Differentially Expressed Genes Correlated With RUNX Genes in Breast Cancer

**FIGURE 6:**
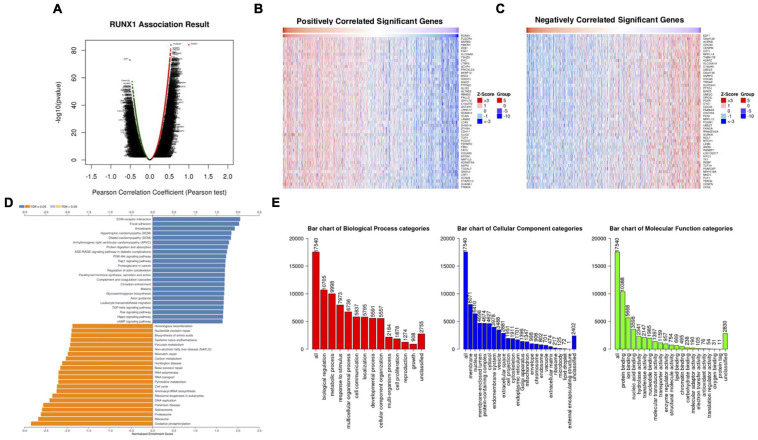
Analysis of differentially expressed genes in correlation with RUNX1 in breast cancer. **(A)** Volcano plot showing the up-regulated and down-regulated genes correlated with RUNX1 expression (Pearson test). The significantly positively correlated **(B)** and negatively correlated **(C)** genes were shown in heatmaps. **(D)** Kyoto Encyclopedia Genes and Genomes (KEGG) pathway analysis of the significantly differentially expressed genes in correlation with RUNX1. **(E)** Gene ontology (GO) analysis of the significantly differentially expressed genes in correlation with RUNX1.

We analyzed the differentially expressed genes that were correlated with RUNX1 in breast cancer using LinkedOmics (Figure 6A). The top 50 positively and 50 negatively correlated genes were visualized in heatmaps (Figures 6B,C). GSEA was performed to identify the enriched KEGG pathways for the top significantly differentially expressed genes (Figure 6D). Among them, ECM-receptor interaction and focal adhesion were significantly upregulated, whereas cell cycle, RNA transport, pyrimidine metabolism, and DNA replication pathways were significantly downregulated. These genes were also classified using GO (Figure 6E). The top three enriched biological process terms were biological regulation, metabolism, and responses to stimulus. The top three enriched cellular component terms were the membrane, nucleus, and membrane-enclosed lumen. The top three enriched molecular function terms were protein binding, ion binding, and nucleic acid binding. Similarly, differentially expressed genes correlated with RUNX2 (Supplementary Figure 4) and RUNX3 (Supplementary Figure 5) were also analyzed and enriched.

### Runt-Related Transcription Factor 1 Promoter Methylation Level in Breast Cancer

**FIGURE 7:**
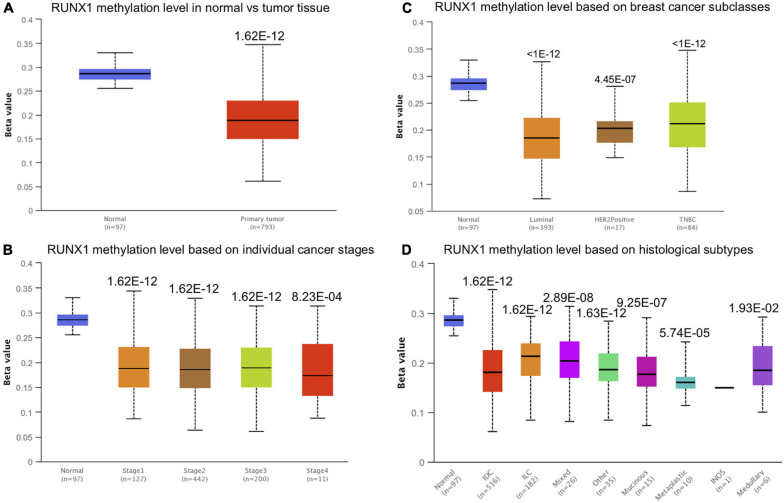
Correlation analysis between RUNX1 methylation level and breast cancer patient characteristics. The RUNX1 methylation level in breast cancer patients classified by **(A)** sample types, **(B)** individual cancer stages, **(C)** breast cancer subclasses, and **(D)** histological subtypes were shown.

**FIGURE 8:**
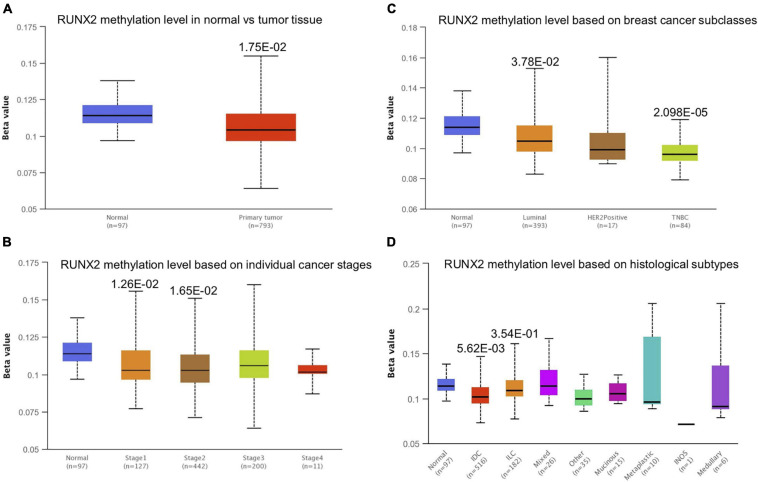
Correlation analysis between RUNX2 methylation level and breast cancer patient characteristics. The RUNX2 methylation level in breast cancer patients classified by **(A)** sample types, **(B)** individual cancer stages, **(C)** breast cancer subclasses, and **(D)** histological subtypes were shown.

**FIGURE 9:**
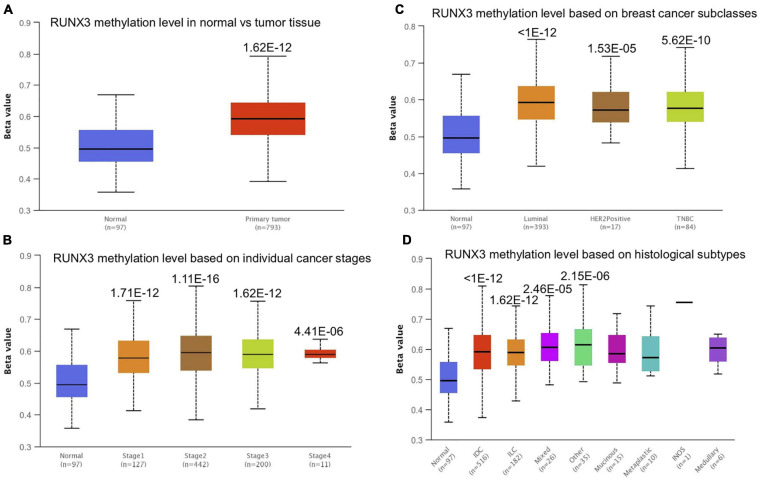
Correlation analysis between RUNX3 methylation level and breast cancer patient characteristics. The RUNX3 methylation level in breast cancer patients classified by **(A)** sample types, **(B)** individual cancer stages, **(C)** breast cancer subclasses, and **(D)** histological subtypes were shown.

Promoter methylation is an important regulator of gene expression. A previous study has shown that promoter methylation contributes to RUNX3 inactivation in breast cancer (Lau et al., 2006). We performed UALCAN analysis to evaluate RUNX1 promoter methylation levels and its relationship with breast cancer patient characteristics. Interestingly, we found that the RUNX1 promoter methylation level was significantly lower in breast cancer tissues than in normal tissues, regardless of stage, subclass, or histological type (Figure 7). This was consistent with the increased RUNX1 expression in breast cancer. Meanwhile, we also found that the RUNX2 promoter methylation level was significantly lower in breast cancer tissues than normal tissues, regardless of stage, subclass, or histological type (Figure 8). In contrast, RUNX3 promoter methylation level was significantly higher in breast cancer tissues than normal tissues, regardless of stage, subclass, or histological type (Figure 9). Moreover, we used the MEXPRESS database to further verify the methylation level of the RUNX family in breast cancer. The results of the MEXPRESS database analysis were consistent with the UALCAN database analysis. These results indicate that different changes in the methylation levels of RUNX factors in breast cancer may account for the distinct effects of RUNX proteins on patient outcome.

### Relationship Between the Expression Level of RUNX Factors and the Abundance of Immune Infiltrates in Breast Cancer

**FIGURE 10:**
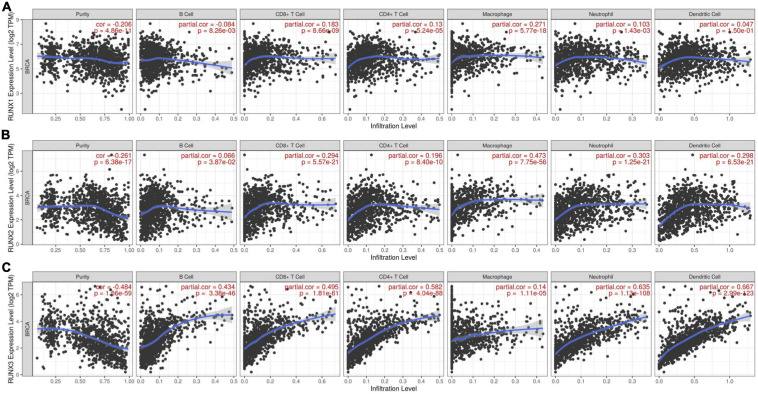
The correlation between differentially expressed RUNXs and immune cell infiltration in breast cancer. The correlations between the transcriptional levels of **(A)** RUNX1, **(B)** RUNX2, and **(C)** RUNX3 with the infiltration of B cells, CD8^+^ T cells, CD4^+^ T cells, macrophages, neutrophils, and dendritic cells in breast cancer were shown.

Runt-related transcription factors can regulate the development and maturation of immune cells, but the relationship between the expression levels of RUNX factors and immune cell infiltration in breast cancer is unclear. Therefore, we used the TIMER database to analyze the expression levels of RUNX factors and their relationship with the infiltration of six types of immune cells (B cells, CD8^+^T cells, CD4^+^T cells, macrophages, neutrophils, and dendritic cells) in breast cancer. The results indicated that the expression of RUNX1 was positively correlated with the infiltration of CD8^+^T cells (COR = 0.183, *P* = 8.66E−09), CD4^+^T cells (COR = 0.13, *P* = 5.24E−05), neutrophils (COR = 0.103, *P* = 1.43E−03), and macrophages (COR = 0.271, *P* = 5.77E−18), whereas it was negatively correlated with B cell infiltration (COR = −0.084, *P* = 8.26E−03) (Figure 10A). The expression level of RUNX2 was positively correlated with the infiltration of all types of immune cells that were analyzed: B cells (COR = 0.066, *P* = 3.87E−02), CD8^+^T cells (COR = 0.294, *P* = 5.57E−21), CD4^+^T cells (COR = 0.196, *P* = 8.40E−10), macrophages (COR = 0.473, *P* = 7.75E−56), neutrophils (COR = 0.303, *P* = 1.25E−21), and dendritic cells (COR = 0.298, *P* = 6.53E−21) (Figure 10B). The expression of RUNX3 was also positively correlated with the infiltration of B cells (COR = 0.434, *P* = 3.38E−46), CD8^+^T cells (COR = 0.495, *P* = 1.81E−61), CD4^+^T cells (COR = 0.582, *P* = 4.04E−88), macrophages (COR = 0.14, *P* = 1.1E−05), neutrophils (COR = 0.635, *P* = 1.13E−108), and dendritic cells (COR = 0.667, *P* = 2.99E−123) (Figure 10C).

Furthermore, we analyzed the relationship between the expression of RUNX1 and the infiltration of six types of immune cells in BRCA-basal, BRCA-HER2, and BRCA-luminal breast cancer subtypes. We found that RUNX1 expression was only positively correlated with CD4^+^T cell invasion (COR = 0.227, *P* = 1.21E−02) in the BRCA-basal type. For the BRCA-HER2 subtype, RUNX1 expression was only positively correlated with macrophage infiltration (COR = 0.29, *P* = 2.72e−02). However, the expression level of RUNX1 in the BRCA-luminal subtype was positively correlated with the infiltration of CD8^+^T cells (COR = 0.161, *P* = 1.84E−04), CD4^+^T cells (COR = 0.208, *P* = 1.15E−06), macrophages (COR = 0.212, *P* = 6.26E−07), neutrophils (COR = 0.192, *P* = 7.81E−06), and dendritic cells (COR = 0.131, *P* = 2.39E−03) (Supplementary Figure 6). We also analyzed the relationship between the expression levels of RUNX2/RUNX3 and the infiltration of immune cells in BRCA-basal, BRCA-HER2, and BRCA-luminal breast cancer subtypes (Supplementary Figures 7, 8).

### Relationship Between RUNX Mutations and Immune Cell Infiltration in Breast Cancer

Various genes are mutated during breast cancer pathogenesis. Through analysis of the TIMER database, we found that RUNX1 had highly frequent mutations in breast cancer, while RUNX2 and RUNX3 had a lower frequency of mutation rate. Therefore, we analyzed the relationship between RUNX1 mutations and the infiltration of immune cells in breast cancer. The results showed that RUNX1 mutations significantly increased the levels of CD8^+^T cells, CD4^+^T cells, and macrophage infiltration in breast cancer (Supplementary Figure 9). This suggests that RUNX1 mutation may be used as a potential marker for detecting immune cell infiltration in breast cancer.

### Correlation Between Gene Copy Number of RUNX Factors and Immune Cell Infiltration

**FIGURE 11:**
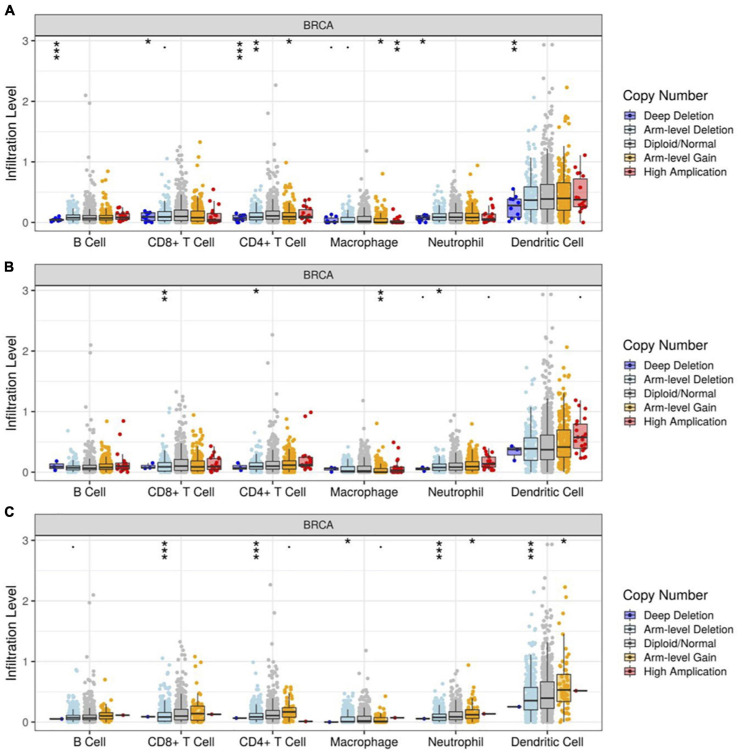
Correlation between RUNX gene copy number and immune cell infiltration in breast cancer. The correlation between the gene copy number changes of **(A)** RUNX1, **(B)** RUNX2, and **(C)** RUNX3 with infiltration levels of B cells, CD8^+^ T cells, CD4^+^ T cells, macrophages, neutrophils, and dendritic cells in breast cancer were shown. *p < 0.05; **p < 0.01; and ***p < 0.001.

Runt-related transcription factors not only affect breast cancer cells, but also modulate the function of immune cells. Through TIMER analysis, we found a positive correlation between RUNX1 gene copy number and the infiltration of B cells, CD8^+^T cells, CD4^+^T cells, macrophages, neutrophils, and dendritic cells in breast cancer (Figure 11A). The copy number change of the RUNX2 gene was only positively correlated with the infiltration of four types of immune cells in breast cancer: CD8^+^T cells, CD4^+^T cells, macrophages, and neutrophils (Figure 11B). In addition, the change in RUNX3 gene copy number was significantly correlated with the infiltration of CD8^+^T cells, CD4^+^T cells, macrophages, neutrophils, and dendritic cells (Figure 11C). These results suggest that changes in the copy number of RUNX genes may reflect the infiltration of multiple immune cells in breast cancer.

Furthermore, we analyzed the relationship between RUNX gene copy number and immune cell infiltration in breast cancer subtypes. We found that the infiltration of CD4^+^T cells, macrophages, and neutrophils in BRCA-basal and BRCA-luminal subtypes was affected by changes in the RUNX1 gene copy number. However, the infiltration of immune cells into the BRCA-HER2 subtypes was not affected. In addition, the change in RUNX1 gene copy number in BRCA-luminal cells also affected the infiltration of B cells (Supplementary Figure 10). The change in RUNX2 gene copy number influenced the infiltration of multiple immune cells in the subtypes. In the BRCA-basal type, the RUNX2 gene copy number was associated with the infiltration of CD8^+^T cells, CD4^+^T cells, and neutrophils. The change in RUNX2 gene copy number in the BRCA-HER2 subtype was correlated with the infiltration of B cells, CD8^+^T cells, CD4^+^T cells, macrophages, neutrophils, and dendritic cells. However, in the BRCA-luminal subtype, the change in RUNX2 gene copy number was only associated with the infiltration of B cells and CD4^+^T cells (Supplementary Figure 11). For RUNX3, changes in gene copy number only affected the infiltration of CD4^+^T cells in BRCA-HER2 and BRCA-luminal subtypes, whereas multiple types of immune cells were affected in the BRCA-basal subtype (Supplementary Figure 12). These results suggest that the change in RUNX1 gene copy number might mainly affect immune cell infiltration in BRCA basal and BRCA-luminal subtypes; the change in RUNX2 gene copy number might mainly affect immune cell infiltration in BRCA-basal and BRCA-HER2 subtypes; whilst the RUNX3 gene copy number change might mainly influence the change in immune cell infiltration in BRCA-basal type.

### Relationship Between RUNX Gene Methylation and Immune Cell Infiltration

**FIGURE 12:**
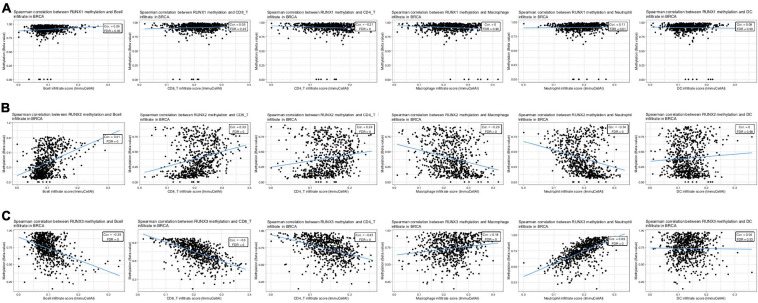
Correlation between RUNX methylation level and immune cell infiltration in breast cancer. The correlations between changes in the methylation levels of **(A)** RUNX1, **(B)** RUNX2, and **(C)** RUNX3 with the infiltration of B cells, CD8^+^ T cells, CD4^+^ T cells, macrophages, neutrophils, and dendritic cells in breast cancer were shown.

Through the above analysis, we have shown the methylation level change of RUNX factors in breast cancer, as well as the relationship between RUNX factor expression level and immune cell infiltration in breast cancer. However, it remains unclear whether changes in the methylation of RUNX genes also influence the infiltration of immune cells in breast cancer. Therefore, we further analyzed the association between the methylation levels of RUNX genes and immune cell infiltration (B cells, CD8^+^T cells, CD4^+^T cells, macrophages, neutrophils, and dendritic cells) in breast cancer using the GSCA database (Figure 12). The results showed that RUNX2 methylation was positively correlated with the infiltration levels of B cells, CD8^+^T cells, and CD4^+^T cells, and negatively correlated with the infiltration levels of macrophages and neutrophils (Figure 12B). In contrast, the methylation level of RUNX3 was negatively correlated with the infiltration of B cells, CD8^+^T cells, and CD4^+^T cells, whereas it was positively correlated with the infiltration levels of macrophages and neutrophils (Figure 12C). This suggests that the methylation levels of RUNX factors may have opposite effects on the same type of immune cell infiltration.

### Correlation Between RUNX Methylation Level and TGF-β1 Expression in Breast Cancer

**FIGURE 13:**
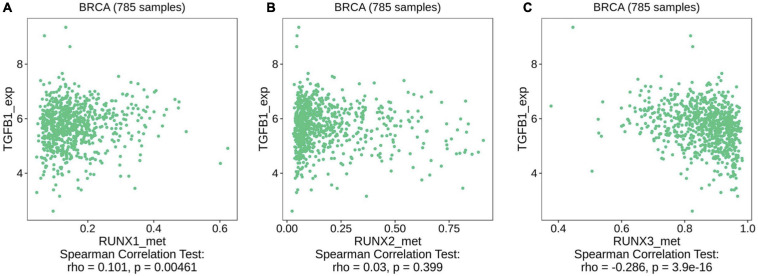
Correlation between RUNX methylation level and TGF-β1 expression in breast cancer. The correlations between changes in the methylation levels of **(A)** RUNX1, **(B)** RUNX2, and **(C)** RUNX3 with the expression of TGF-β1 in breast cancer were shown.

TGF-β is a critical negative immunoregulatory factor in immune balance. TGF-β not only inhibits various targets in the immune system, but also plays a role in tumor immune escape and adverse reactions to tumor immunotherapy (Batlle and Massague, 2019; Derynck et al., 2021). Through analysis of the TIMER database, we found that the expression of TGFB1, TGFBR1, and TGFBR2 are correlated with the infiltration levels of various immune cells in breast cancer (Supplementary Figure 13). We used the TIMER database to further analyze the relationship between the expression level of TGFB1 and immune cell infiltration in different breast cancer subtypes. We found that the expression level of TGFB1 was positively correlated with the infiltration level of various immune cells in different breast cancer subtypes (Supplementary Figure 14). The relationship between the expression levels of TGFBR1 and TGFBR2 and immune cell infiltration in different breast cancer subtypes was also analyzed. We found that the expression level of TGFBR1 was mainly correlated with the level of macrophage infiltration in different breast cancer subtypes (Supplementary Figure 15). The expression level of TGFBR2 was positively correlated with the infiltration level of multiple immune cells in different subtypes of breast cancer (Supplementary Figure 16). These results indicate that the TGF-β signaling pathway is related to the infiltration level of multiple immune cells in different subtypes of breast cancer. However, it remains unclear whether the methylation levels of RUNX genes affect the TGF-β signaling pathway. To further evaluate the potential mechanisms underlying the effects of RUNX gene methylation on immune cell infiltration in breast cancer, we analyzed the relationship between the methylation level of RUNX genes and the TGF-β signaling pathway using the TISIDB database. The results showed that RUNX1 gene methylation level was positively correlated with the expression level of TGF-β1 (Figure 13A), while there was no correlation between RUNX2 methylation level and the expression of TGF-β1 (Figure 13B). However, RUNX3 methylation levels were negatively correlated with the expression levels of TGF-β1 (Figure 13C). The relationship between the methylation level of RUNX genes and the expression of TGFBR1 was also analyzed, but no correlation was identified (Supplementary Figure 17). These results suggest that changes in the methylation level of RUNX genes may affect the expression of TGF-β1, which in turn might influence the activation of the TGF-β signaling pathway and immune cell infiltration in breast cancer.

## Discussion

The pathophysiology of breast cancer is multifaceted and is affected by both genetic and environmental factors. However, the clinical relevance of the immune system in breast cancer has not been adequately studied. The heterogeneity of breast cancer has been highlighted in the past decade, and this has stimulated the exploration of the relationship between the immune system and heterogeneity in different breast cancer subtypes. An increasing number of studies have shown that there are multiple immune cell infiltrates in breast cancer tissue, which could affect patient outcomes. These immune cells affect the pathogenesis and metastasis of breast cancer through various signaling pathways. For example, neutrophils can promote lung metastasis of breast cancer by secreting leukotriene B4 (Wculek and Malanchi, 2015), whereas podoplanin-expressing macrophages can contribute to the lymphoinvasion of breast cancer cells by binding with lymphatic endothelial cells to trigger matrix remodeling and lymphangiogenesis (Bieniasz-Krzywiec et al., 2019). In addition, lymphocyte infiltration in breast cancer is associated with clinical survival outcomes (Savas et al., 2016; Byrne et al., 2020). Although the essential role of immune cell infiltration in breast cancer has gained widespread attention, the relationship between the RUNX family and immune cell infiltration remains poorly understood.

An increasing number of alterations have been identified in multiple tumor suppressor or activator genes in breast cancer patients, among which the RUNX1 gene has a high frequency of alterations. RUNX1 is a member of the RUNX family, which consists of RUNX1, RUNX2, and RUNX3 in mammals. They affect a variety of signaling pathways, including Wnt (Ito et al., 2008), Notch (Hilton et al., 2008), MST-YAP1 (Min et al., 2012), and receptor tyrosine kinase (Huang H. et al., 2012). These signaling pathways are of great importance during multiple stages of breast cancer pathogenesis and metastasis.

Multiple mechanisms have been studied to elucidate the role of the RUNX family in breast cancer. RUNX1 has been shown to inhibit the growth of breast cancer stem cells and promote tumor expansion (Hong et al., 2018). In addition, RUNX1 and RUNX3 may inhibit the YAP-regulated epithelial-mesenchymal transition process and improve breast cancer outcome (Kulkarni et al., 2018). In contrast, RUNX2 has been shown to increase the bone metastasis of breast cancer in an integrin-dependent way (Li X.Q. et al., 2016). However, a comprehensive analysis of the transcriptional changes in RUNX family members, promoter methylation level, RUNX gene alternation, and their relationship with immune cell infiltration as well as breast cancer patient prognosis, is still lacking.

Runt-related transcription factor members are critical during the regulation of immune cell development and function. For example, the differentiation of CD4^–^CD8^–^ T lymphocytes to CD4^+^CD8^+^ T lymphocytes depends on the expression of RUNX1 (Egawa et al., 2007). RUNX3 can synergistically regulate the transcription of CD8^+^T cells with the T-box protein (Cruz-Guilloty et al., 2009). It is also important to analyze the correlation between RUNX expression levels and the infiltration of various immune cells in breast cancer. Using the TIMER database, we analyzed the correlation between the expression levels of RUNX protein family members and the infiltration of B cells, CD8^+^T cells, CD4^+^T cells, macrophages, neutrophils, and dendritic cells in different breast cancer subtypes. We also found that RUNX1 mutations in breast cancer significantly increased the infiltration of CD8^+^T cells, CD4^+^T cells, and macrophages in breast cancer.

Studies have shown that epigenetic modifications play an important role in the occurrence and development of breast cancer (Hinshelwood and Clark, 2008; Pasculli et al., 2018). It has been reported that promoters of more than 100 genes undergo hypermethylation in breast cancer (Jovanovic et al., 2010). The promoter region of RUNX3 is hypermethylated, resulting in its decreased expression in breast cancer (Jiang et al., 2008; Liu H. et al., 2018). To the best of our knowledge, our study provides the first detailed bioinformatic analysis of RUNX family gene methylation changes and its relationship with breast cancer characteristics, especially immune cell infiltration in breast cancer. We found that the methylation level of RUNX3 in breast cancer is completely opposite to that of RUNX1 and RUNX2. Interestingly, we also found opposite effects of RUNX2 and RUNX3 gene methylation changes on immune cell infiltration in breast cancer. Our findings suggest that changes in RUNX gene methylation may be a potential biomarker for immune cell infiltration in breast cancer.

Dysregulation of the growth factor signaling pathway is a significant characteristic of tumorigenesis and metastasis. The TGF-β signaling pathway not only regulates tumor cells but also modulates immune cells in the tumor microenvironment, thus playing an important role in the above process (Ikushima and Miyazono, 2010; Batlle and Massague, 2019; Derynck et al., 2021). For example, the TGF-β signaling pathway could downregulate the expression of TNF and IFN-γ, thereby inhibiting the proliferation of CD4^+^T cells (Mangan et al., 2006). Through database analysis, we found that the expression levels of TGFB1 and TGFBR1 in breast cancer are correlated with the infiltration levels of multiple immune cells. To date, the relationship between RUNX gene methylation and the TGF-β signaling pathway remains unclear. Through analysis of the TISDB database, we found that the methylation levels of RUNX genes were correlated with the expression level of TGF-β1, which may affect TGF-β signaling pathway activation and immune cell infiltration in breast cancer.

In summary, our analysis deepens our understanding of the role of RUNX factors and TGF-β signaling pathway in breast cancer. This study will aid in elucidating the molecular mechanisms underlying the role of the RUNX family in breast cancer and provide potential therapeutic targets to improve breast cancer patient outcomes.

## Data Availability Statement

The original contributions presented in the study are included in the article/Supplementary Material, further inquiries can be directed to the corresponding author.

## Author Contributions

FZ constructed the study and revised this manuscript. LG performed the data analysis and wrote the manuscript. Both authors read and approved the final manuscript.

## Conflict of Interest

The authors declare that the research was conducted in the absence of any commercial or financial relationships that could be construed as a potential conflict of interest.

## Publisher’s Note

All claims expressed in this article are solely those of the authors and do not necessarily represent those of their affiliated organizations, or those of the publisher, the editors and the reviewers. Any product that may be evaluated in this article, or claim that may be made by its manufacturer, is not guaranteed or endorsed by the publisher.
