# Supplementary material for: Comprehensive Analysis of RUNX and TGF-β Mediated Regulation of Immune Cell Infiltration in Breast Cancer
Source: Front Cell Dev Biol. 2021 Aug 18;9:730380. doi: 10.3389/fcell.2021.730380 (PMC8416425; doi:10.3389/fcell.2021.730380)
Supplement: Supplementary file 1 [file Table_1.docx]

**Supplementary Figure 1. Correlation Analysis Between RUNX2 Expression and Breast Cancer Patient Characteristics.** The RUNX2 expression levels in breast cancer patients classified by (A) TP53 mutation status, (B) individual cancer stages, (C) breast cancer subclasses, and (D) histological subtypes were shown.

**Supplementary Figure 2. Correlation Analysis Between RUNX3 Expression and Breast Cancer Patient Characteristics.** The RUNX3 expression levels in breast cancer patients classified by (A) TP53 mutation status, (B) individual cancer stages, (C) breast cancer subclasses, and (D) histological subtypes were shown.

**Supplementary Figure 3. Locations of RUNX Mutations along the RUNX genes in Breast Cancer**

**Supplementary Figure 4. Analysis of Differentially Expressed Genes in Correlation with RUNX2 in Breast Cancer. (A)** Volcano plot showing the up-regulated and down-regulated genes correlated with RUNX2 expression (Pearson test). The significantly positively correlated **(B)** and negatively correlated**(C)** genes were shown in heatmaps. **(D)** KEGG pathway analysis of the significantly differentially expressed genes in correlation with RUNX2. **(E)** Gene ontology analysis of the significantly differentially expressed genes in correlation with RUNX2.

**Supplementary Figure 5. Analysis of Differentially Expressed Genes in Correlation with RUNX3 in Breast Cancer. (A)** Volcano plot showing the up-regulated and down-regulated genes correlated with RUNX3 expression (Pearson test). The significantly positively correlated **(B)** and negatively correlated**(C)** genes were shown in heatmaps. **(D)** KEGG pathway analysis of the significantly differentially expressed genes in correlation with RUNX3. **(E)** Gene ontology analysis of the significantly differentially expressed genes in correlation with RUNX3.

**Supplementary Figure 6. The Correlation Between RUNX1 Expression and Immune Cell Infiltration in Different Breast Cancer Subtypes.** The correlation between RUNX1 expression and the infiltration of B cell, CD8^+^ T cell, CD4^+^ T cell, macrophage, neutrophil, and dendritic cell in BRCA-basal, BRCA-HER2, and BRCA-luminal breast cancer subtypes.

**Supplementary Figure 7. The Correlation Between RUNX2 Expression and Immune Cell Infiltration in Different Breast Cancer Subtypes.** The correlation between RUNX2 expression and the infiltration of B cell, CD8^+^ T cell, CD4^+^ T cell, macrophage, neutrophil, and dendritic cell in BRCA-basal, BRCA-HER2, and BRCA-luminal breast cancer subtypes.

**Supplementary Figure 8. The Correlation Between RUNX3 Expression and Immune Cell Infiltration in Different Breast Cancer Subtypes.** The correlation between RUNX3 expression and the infiltration of B cell, CD8^+^ T cell, CD4^+^ T cell, macrophage, neutrophil, and dendritic cell in BRCA-basal, BRCA-HER2, and BRCA-luminal breast cancer subtypes.

**Supplementary Figure 9. Correlation Between RUNX1 Mutation and Immune Cell Infiltration Levels in Breast Cancer.** The infiltration levels of B cell, CD8^+^ T cell, CD4^+^ T cell, macrophage, neutrophil, and dendritic cell were compared between breast cancer patients with different RUNX1 mutation status. * p < 0.05.

**Supplementary Figure 10. Correlation Between RUNX1 Gene Copy Number and Immune Cell Infiltration in Breast Cancer Subtypes.** The correlation between RUNX1 gene copy number changes and the infiltration levels of B cell, CD8^+^ T cell, CD4^+^ T cell, macrophage, neutrophil, and dendritic cell in breast cancer subtypes. * p < 0.05; ** p < 0.01; *** p < 0.001.

**Supplementary Figure 11. Correlation Between RUNX2 Gene Copy Number and Immune Cell Infiltration in Breast Cancer Subtypes.** The correlation between RUNX2 gene copy number changes and the infiltration levels of B cell, CD8^+^ T cell, CD4^+^ T cell, macrophage, neutrophil, and dendritic cell in breast cancer subtypes. * p < 0.05; ** p < 0.01.

**Supplementary Figure 12. Correlation Between RUNX3 Gene Copy Number and Immune Cell Infiltration in Breast Cancer Subtypes.** The correlation between RUNX3 gene copy number changes and the infiltration levels of B cell, CD8^+^ T cell, CD4^+^ T cell, macrophage, neutrophil, and dendritic cell in breast cancer subtypes. * p < 0.05; ** p < 0.01.

**Supplementary Figure 13. The Correlation Between TGF-β signaling pathway and Immune Cell Infiltration in Breast Cancer. (A)** The correlations between the expression of TGFB1 with the infiltration of B cell, CD8^+^ T cell, CD4^+^ T cell, macrophage, neutrophil, and dendritic cell in breast cancer were shown. **(B)** The correlations between the expression of TGFBR1 with the infiltration of B cell, CD8^+^ T cell, CD4^+^ T cell, macrophage, neutrophil, and dendritic cell in breast cancer were shown. **(C)** The correlations between the expression of TGFBR2 with the infiltration of B cell, CD8^+^ T cell, CD4^+^ T cell, macrophage, neutrophil, and dendritic cell in breast cancer were shown.

**Supplementary Figure 14. The Correlation Between TGFB1 Expression and Immune Cell Infiltration in Different Breast Cancer Subtypes.** The correlation between TGFB1 expression and the infiltration of B cell, CD8^+^ T cell, CD4^+^ T cell, macrophage, neutrophil, and dendritic cell in BRCA-basal, BRCA-HER2, and BRCA-luminal breast cancer subtypes

**Supplementary Figure 15. The Correlation Between TGFBR1 Expression and Immune Cell Infiltration in Different Breast Cancer Subtypes.** The correlation between TGFBR1 expression and the infiltration of B cell, CD8^+^ T cell, CD4^+^ T cell, macrophage, neutrophil, and dendritic cell cell in BRCA-basal, BRCA-HER2, and BRCA-luminal breast cancer subtypes.

**Supplementary Figure 16. The Correlation Between TGFBR2 Expression and Immune Cell Infiltration in Different Breast Cancer Subtypes.** The correlation between TGFBR2 expression and the infiltration of B cell, CD8^+^ T cell, CD4^+^ T cell, macrophage, neutrophil, and dendritic cell cellin BRCA-basal, BRCA-HER2, and BRCA-luminal breast cancer subtypes.

**Supplementary Figure 17. Correlation Between RUNX Methylation Level and TGFBR1 Expression in Breast Cancer.** The correlations between changes in the methylation levels of **(A)** RUNX1, **(B)** RUNX2, and **(C)** RUNX3 with the expression of TGFBR1 in breast cancer were shown.
