# Supplementary figures and images for: Comprehensive Analysis of RUNX and TGF-β Mediated Regulation of Immune Cell Infiltration in Breast Cancer
Source: Front Cell Dev Biol. 2021 Aug 18;9:730380. doi: 10.3389/fcell.2021.730380 (PMC8416425; doi:10.3389/fcell.2021.730380)

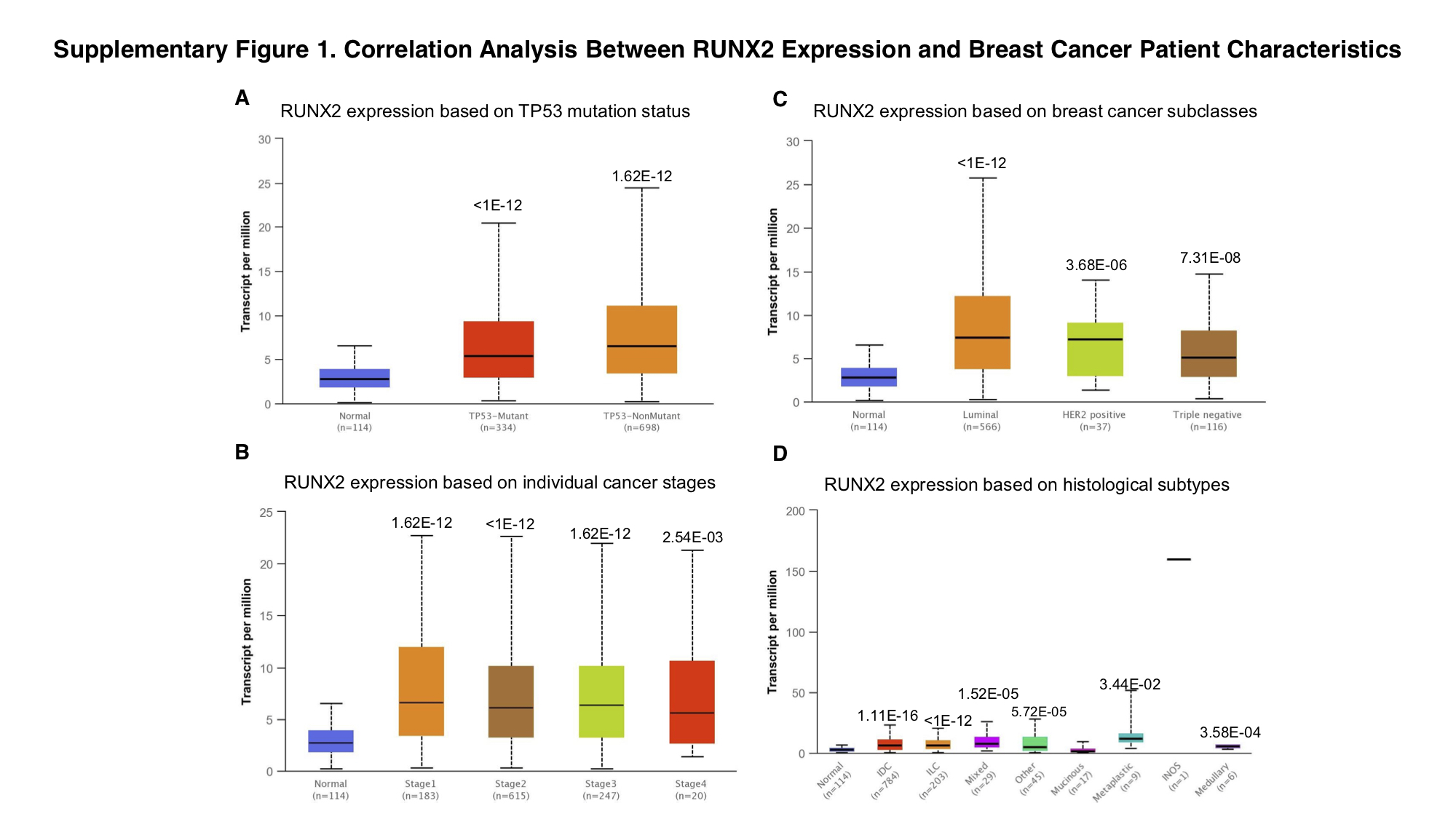

Supplement: Supplementary file 2 [file Image_1.JPEG]

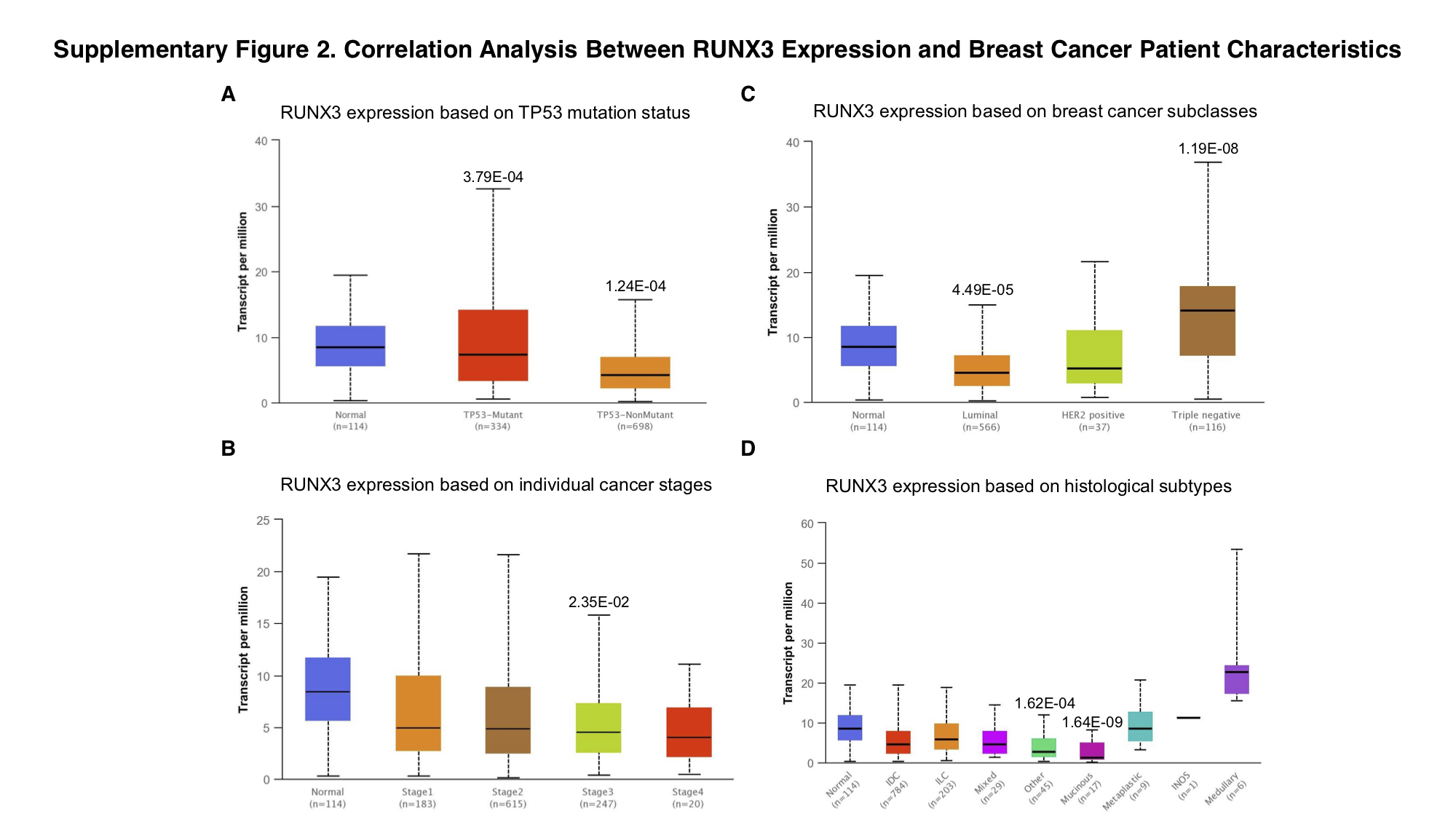

Supplement: Supplementary file 3 [file Image_2.JPEG]

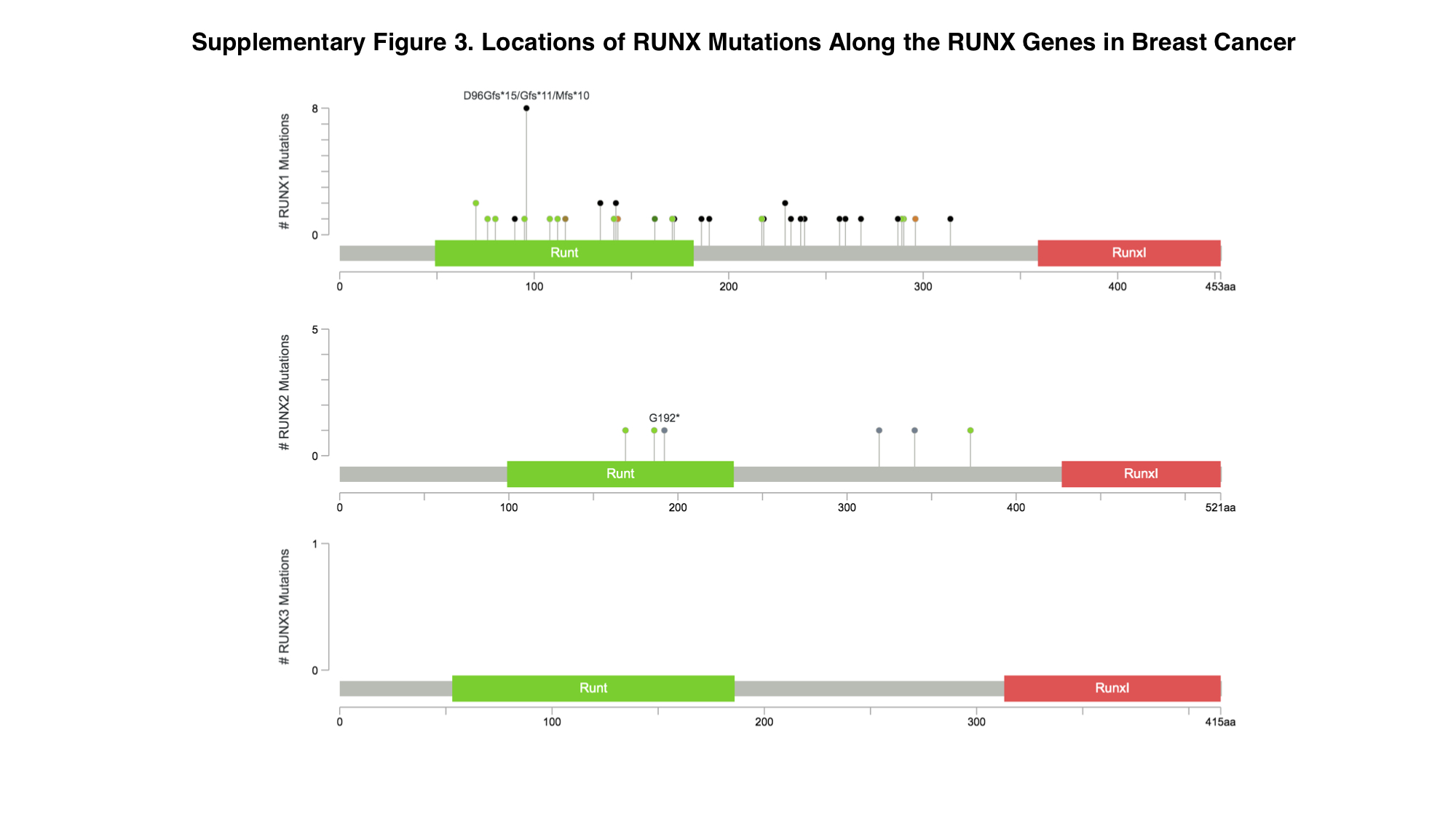

Supplement: Supplementary file 4 [file Image_3.JPEG]

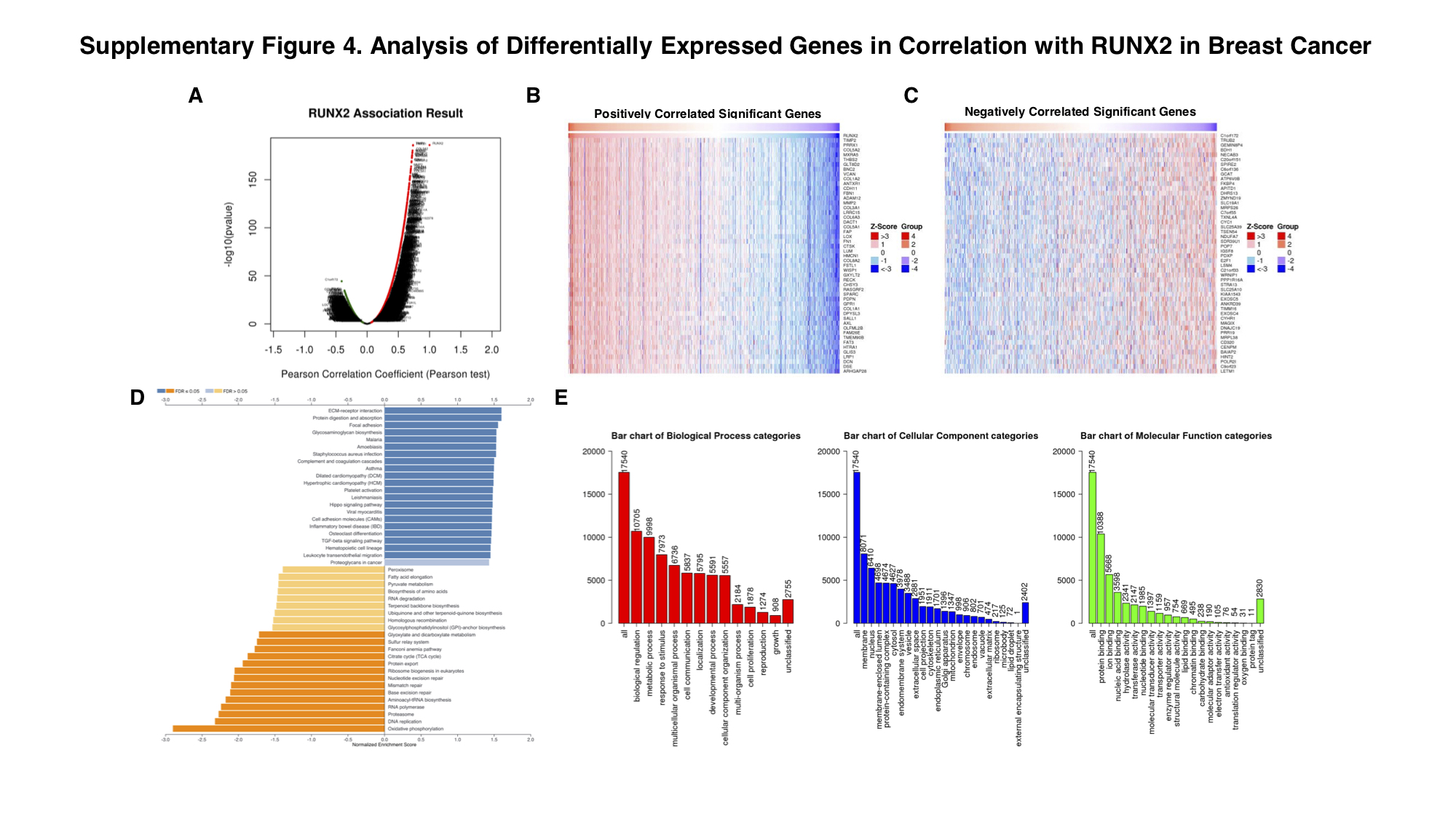

Supplement: Supplementary file 5 [file Image_4.JPEG]

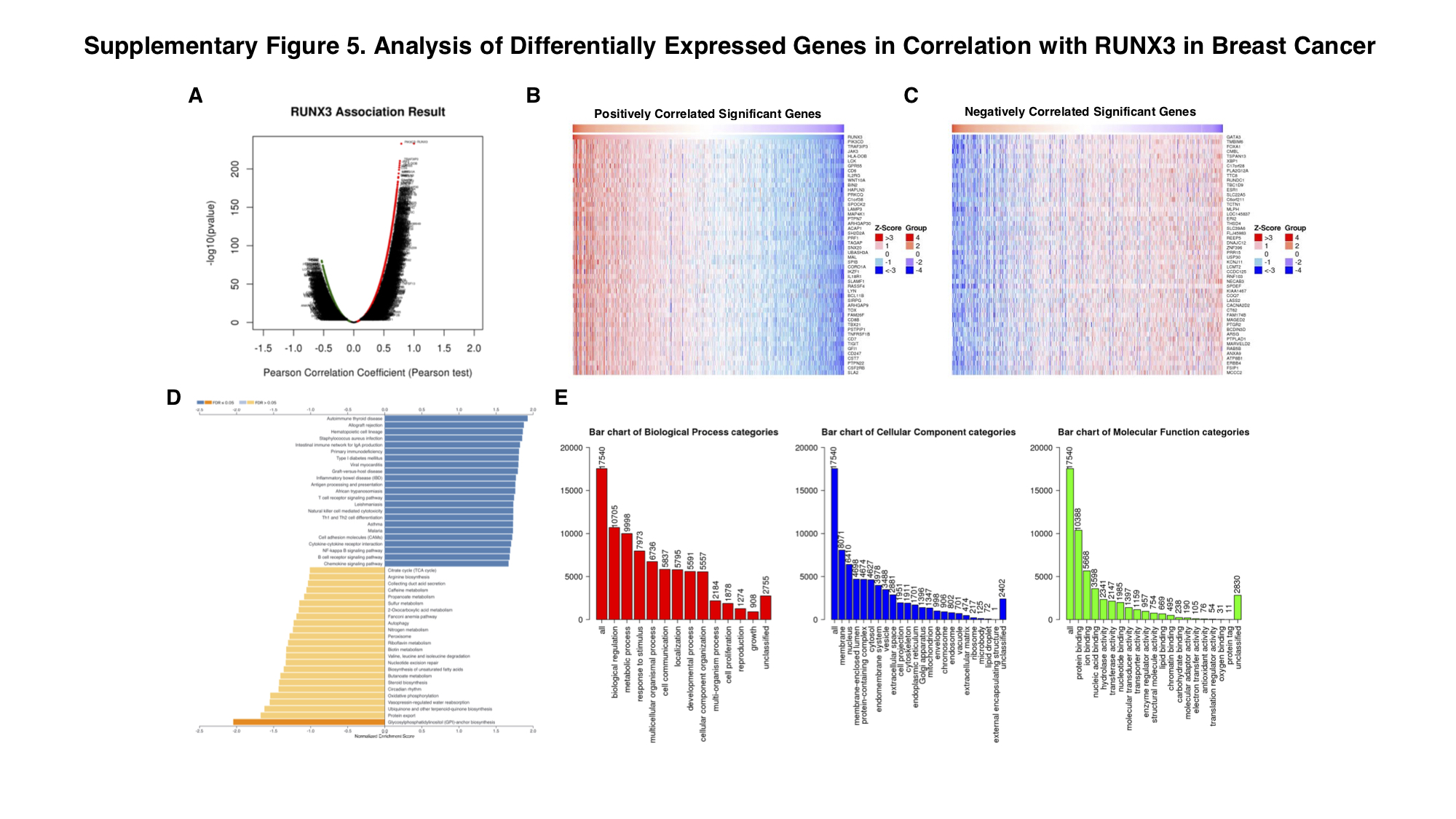

Supplement: Supplementary file 6 [file Image_5.JPEG]

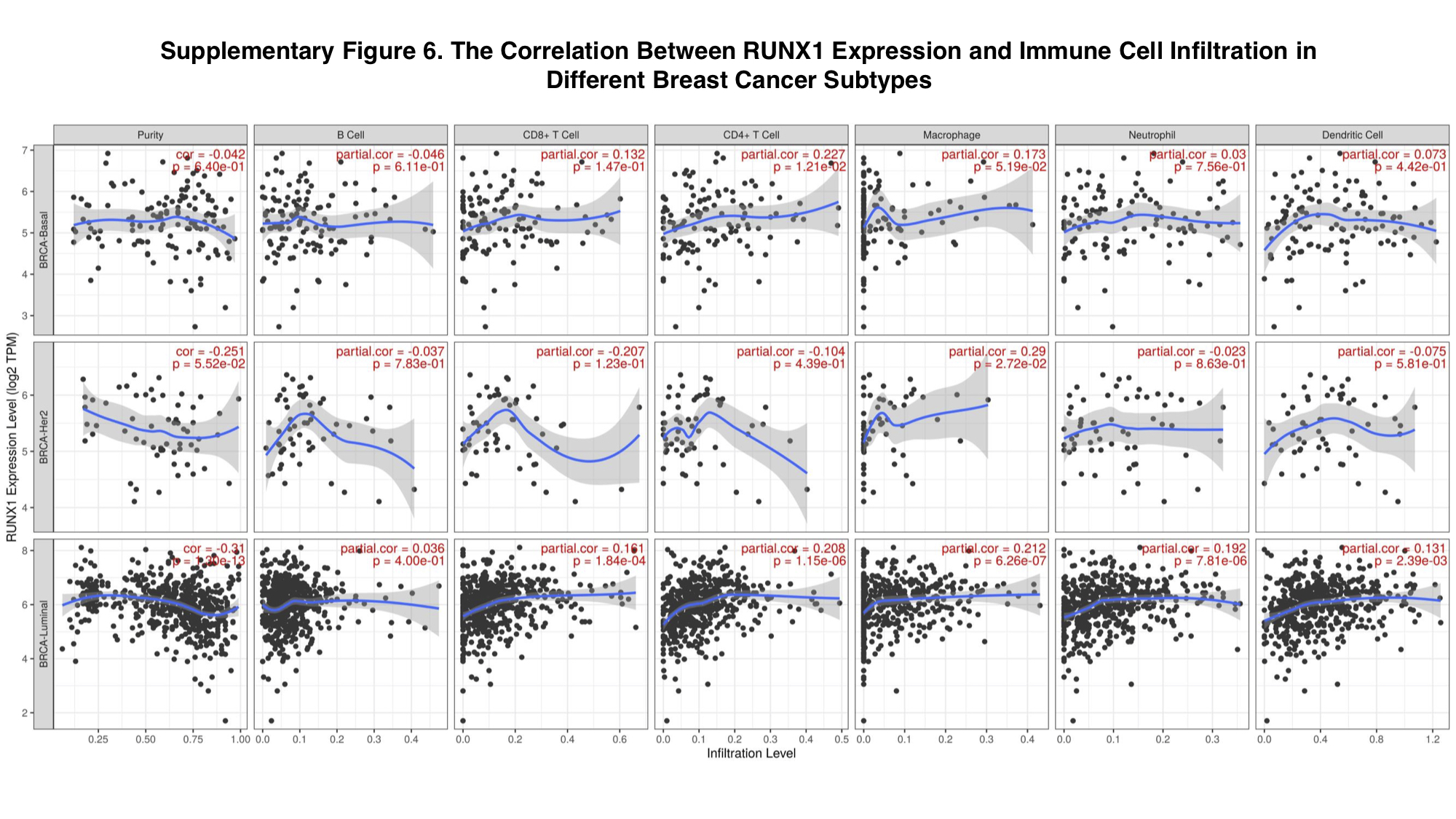

Supplement: Supplementary file 7 [file Image_6.JPEG]

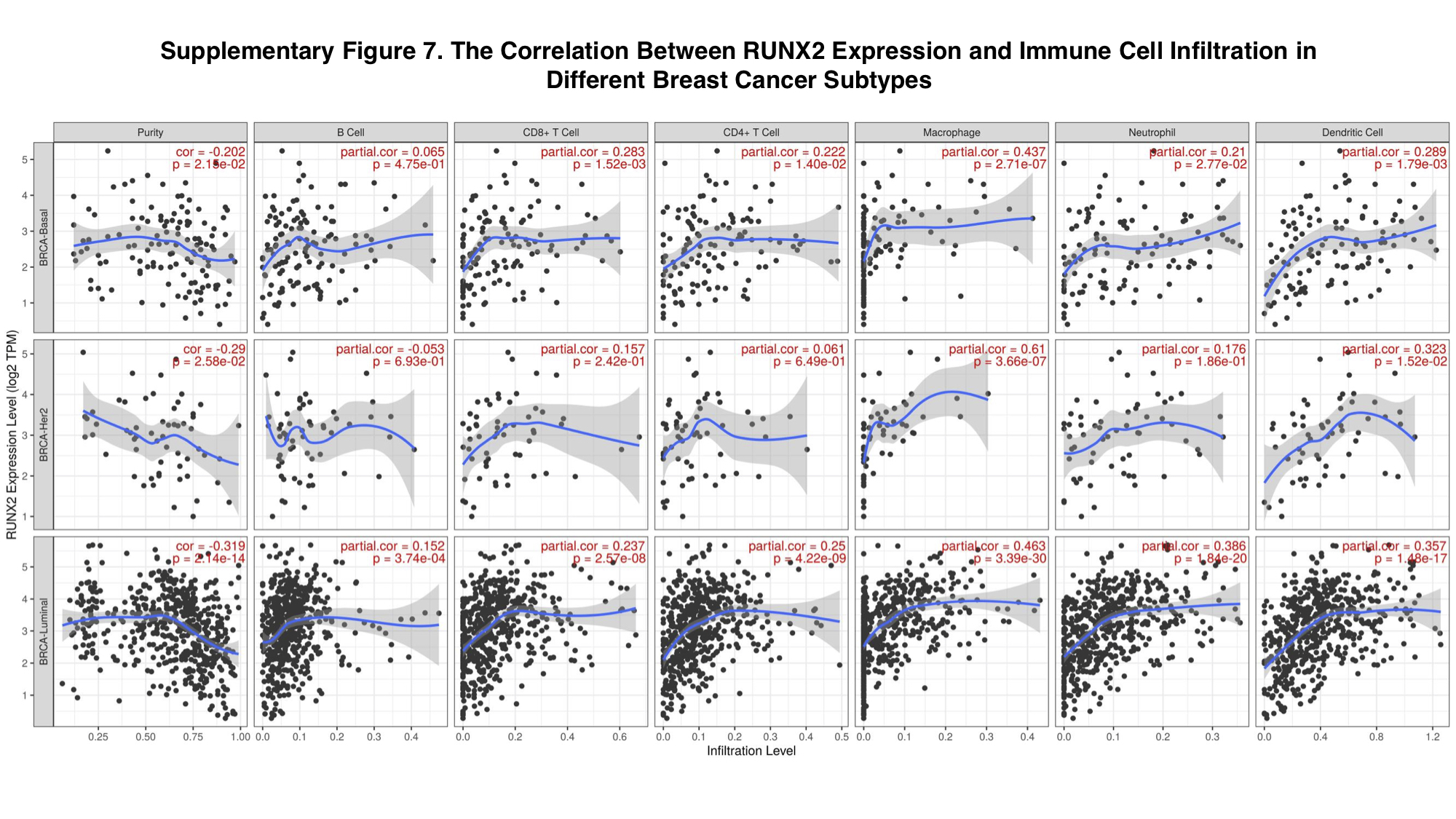

Supplement: Supplementary file 8 [file Image_7.JPEG]

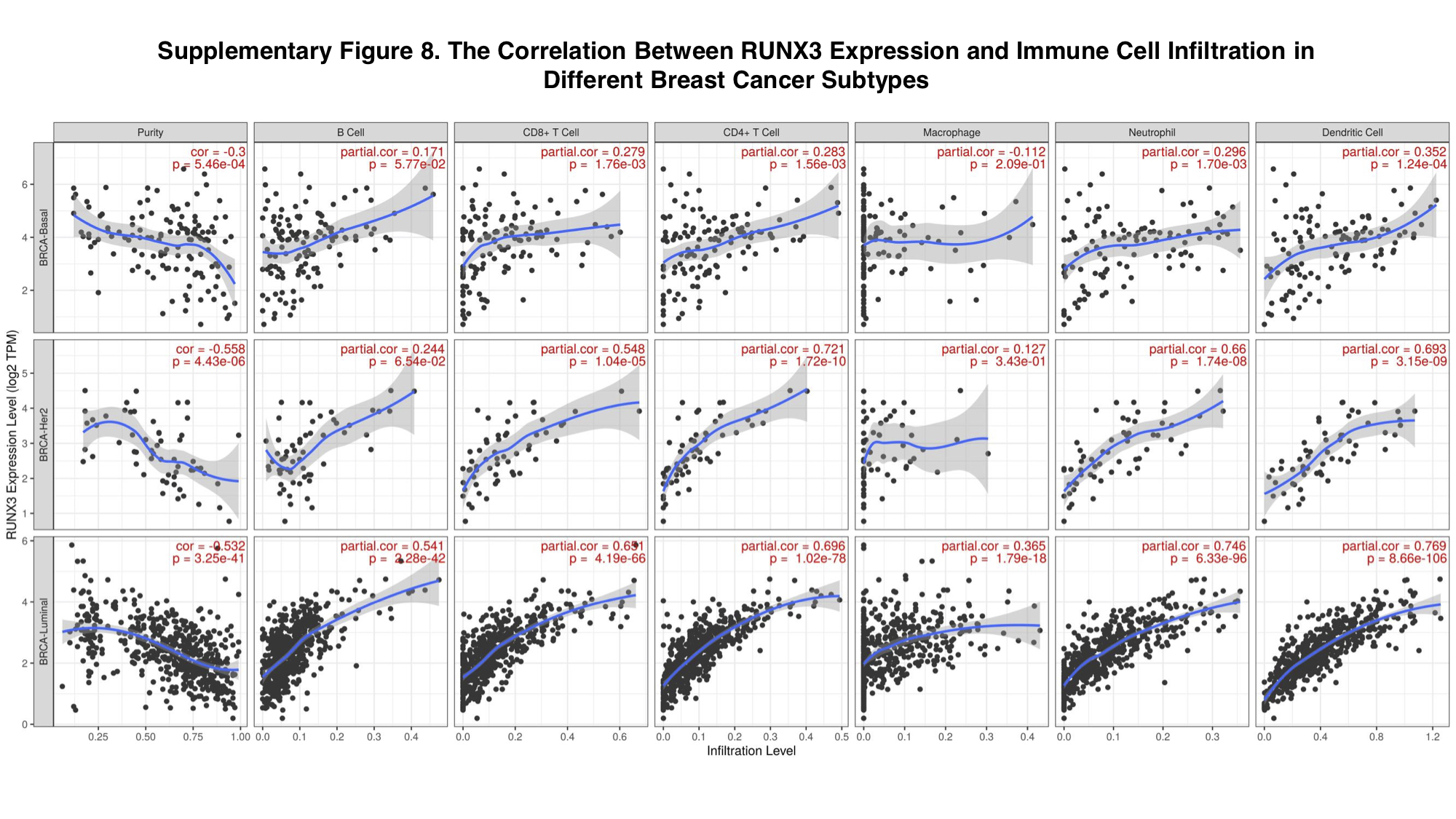

Supplement: Supplementary file 9 [file Image_8.JPEG]

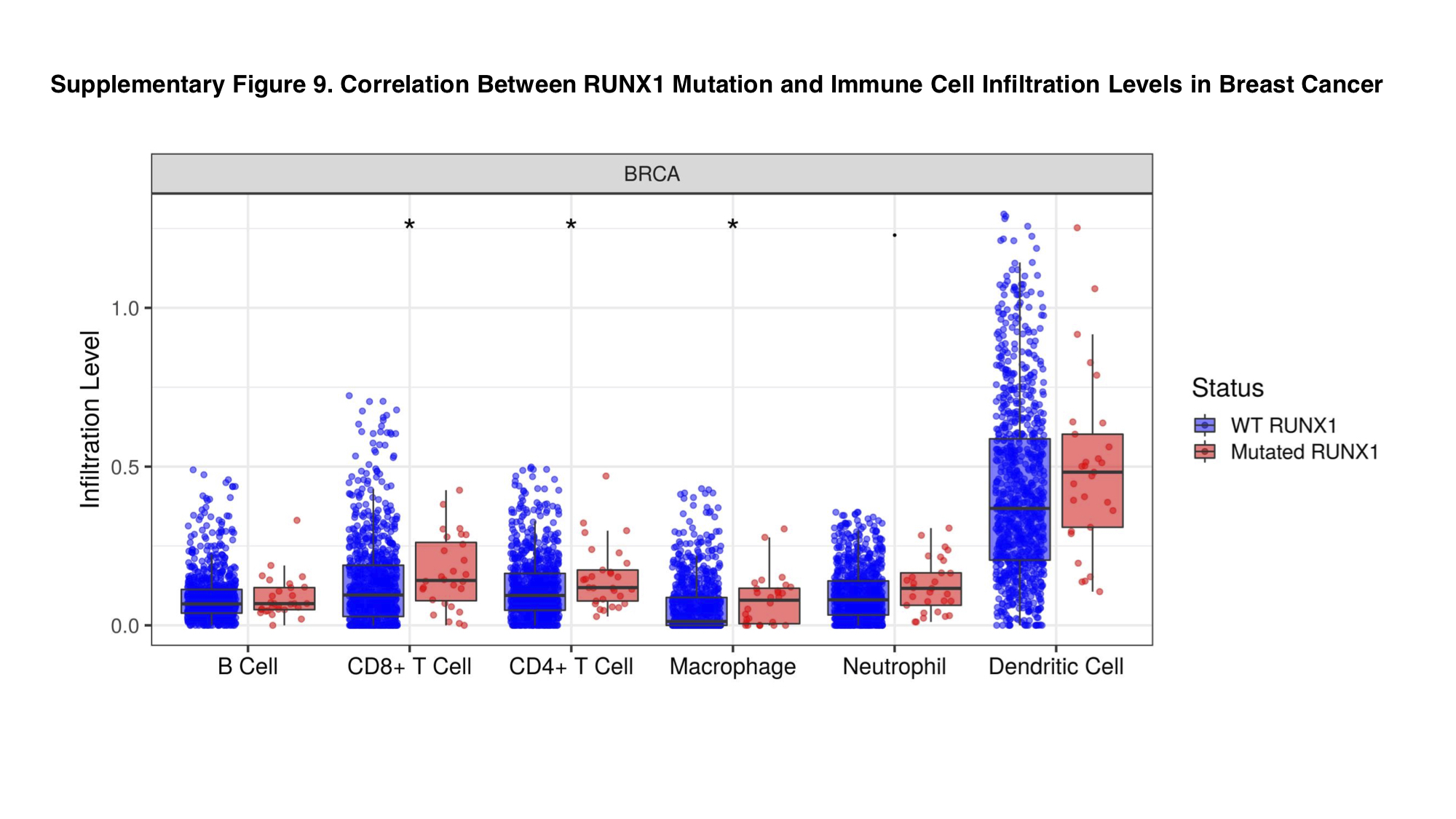

Supplement: Supplementary file 10 [file Image_9.JPEG]

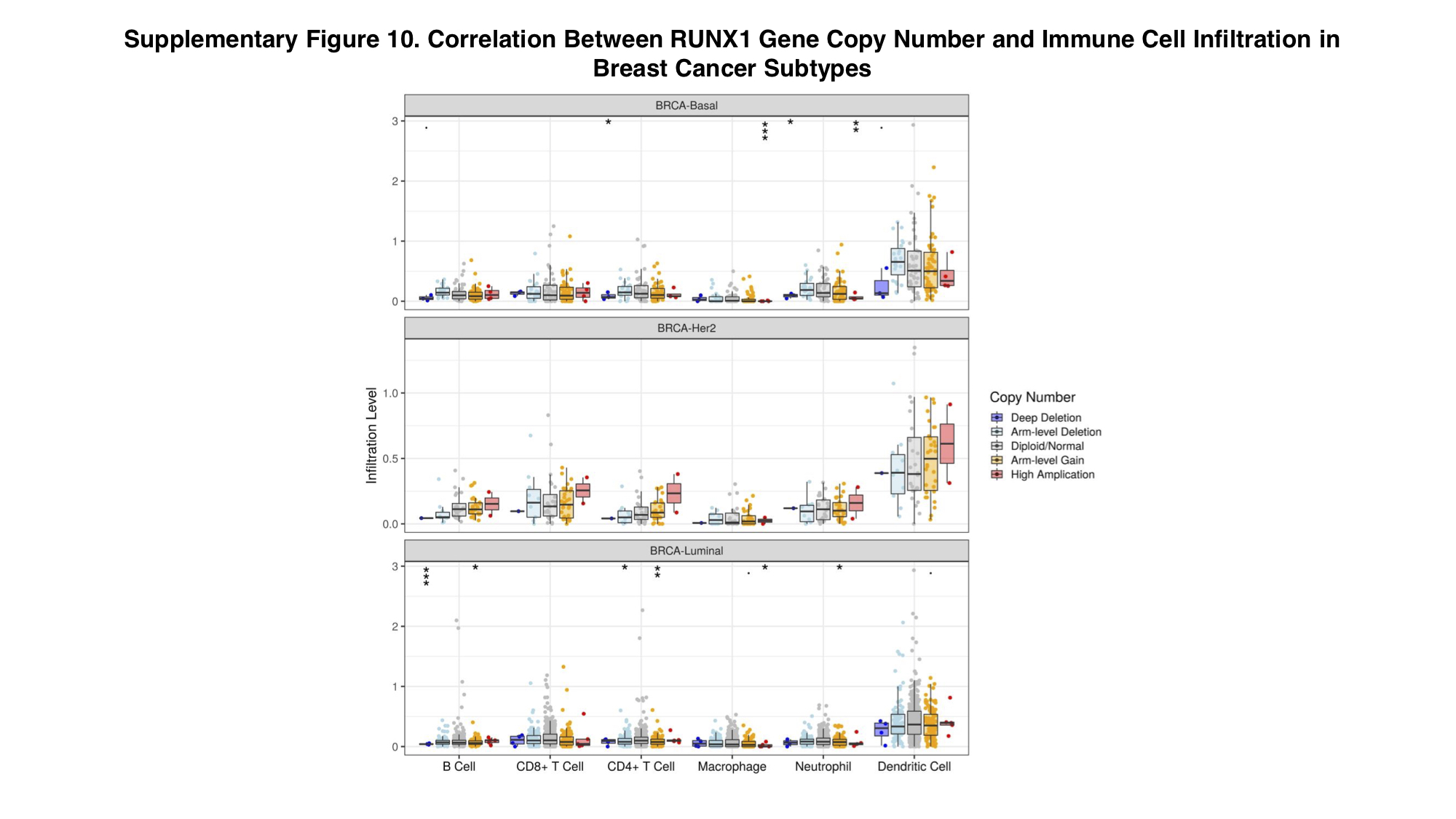

Supplement: Supplementary file 11 [file Image_10.JPEG]

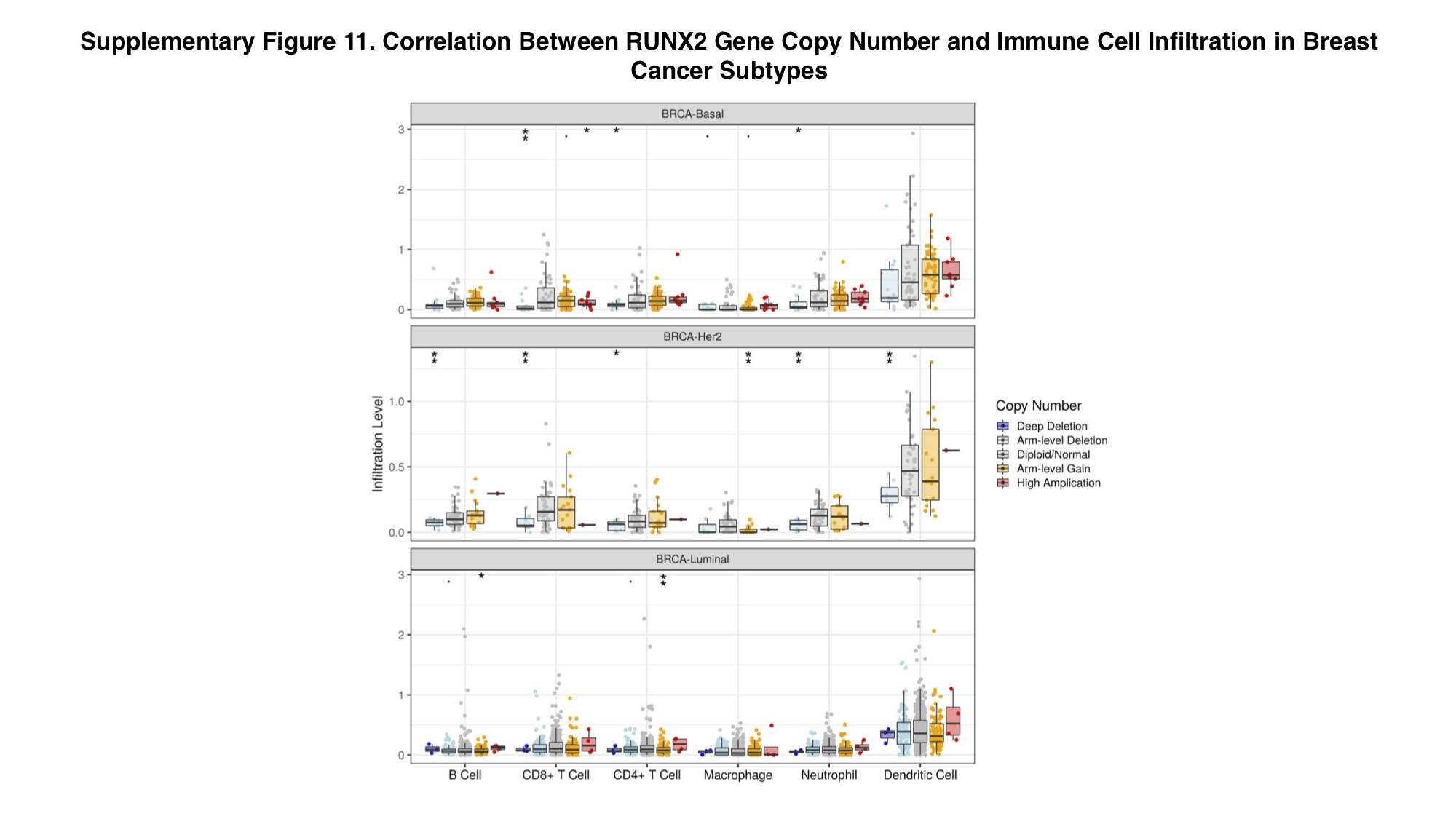

Supplement: Supplementary file 12 [file Image_11.JPEG]

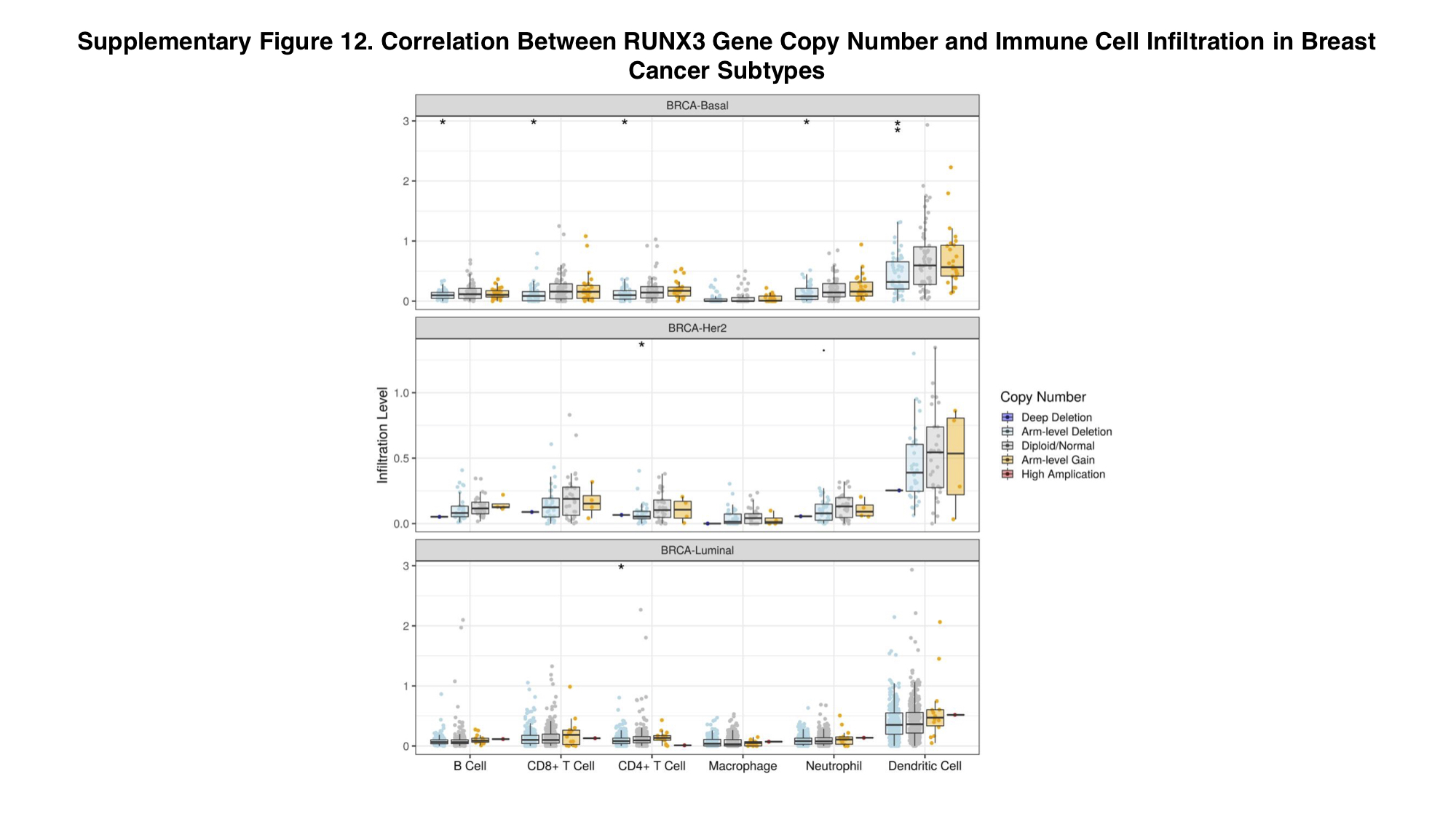

Supplement: Supplementary file 13 [file Image_12.JPEG]

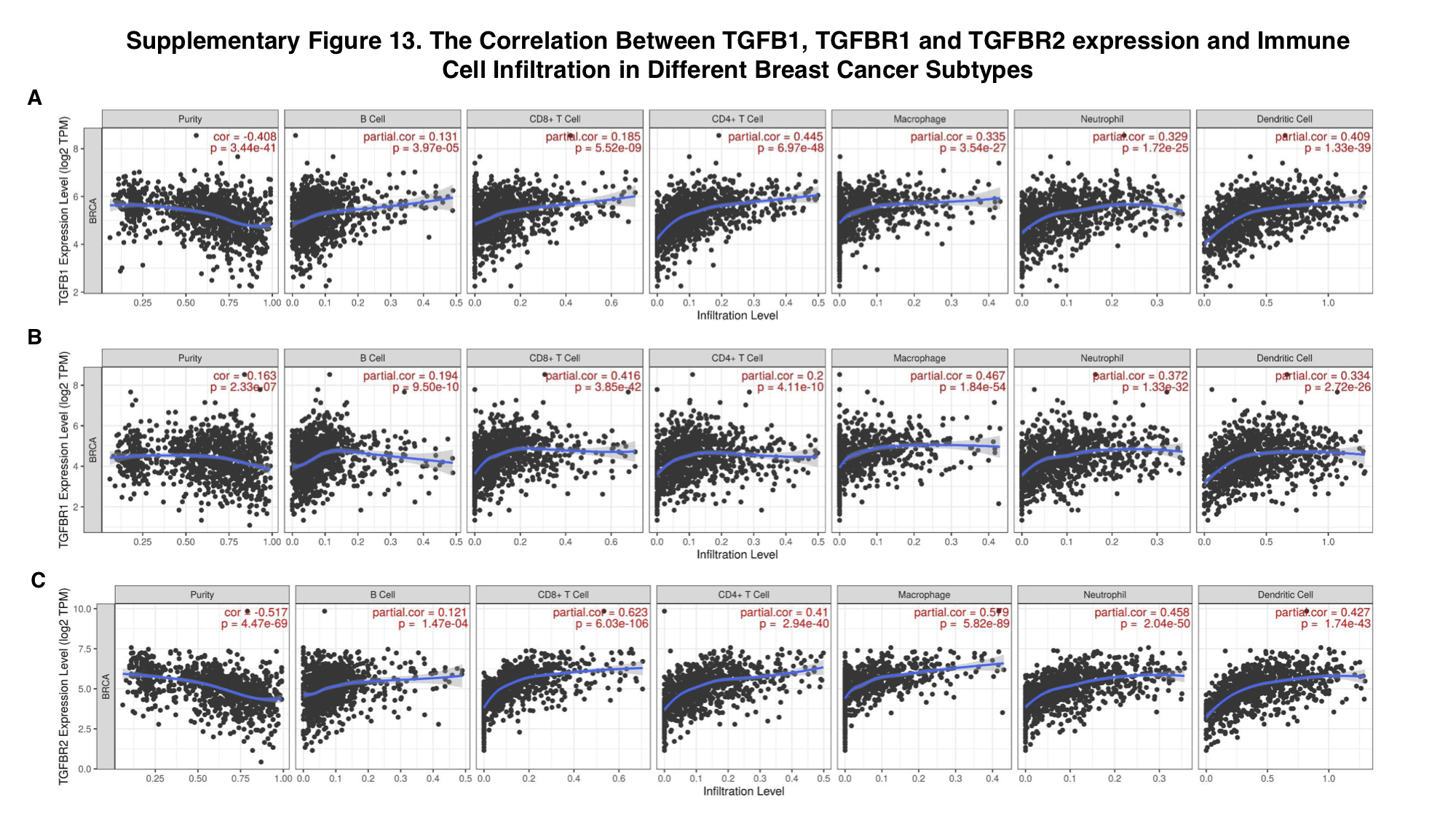

Supplement: Supplementary file 14 [file Image_13.JPEG]

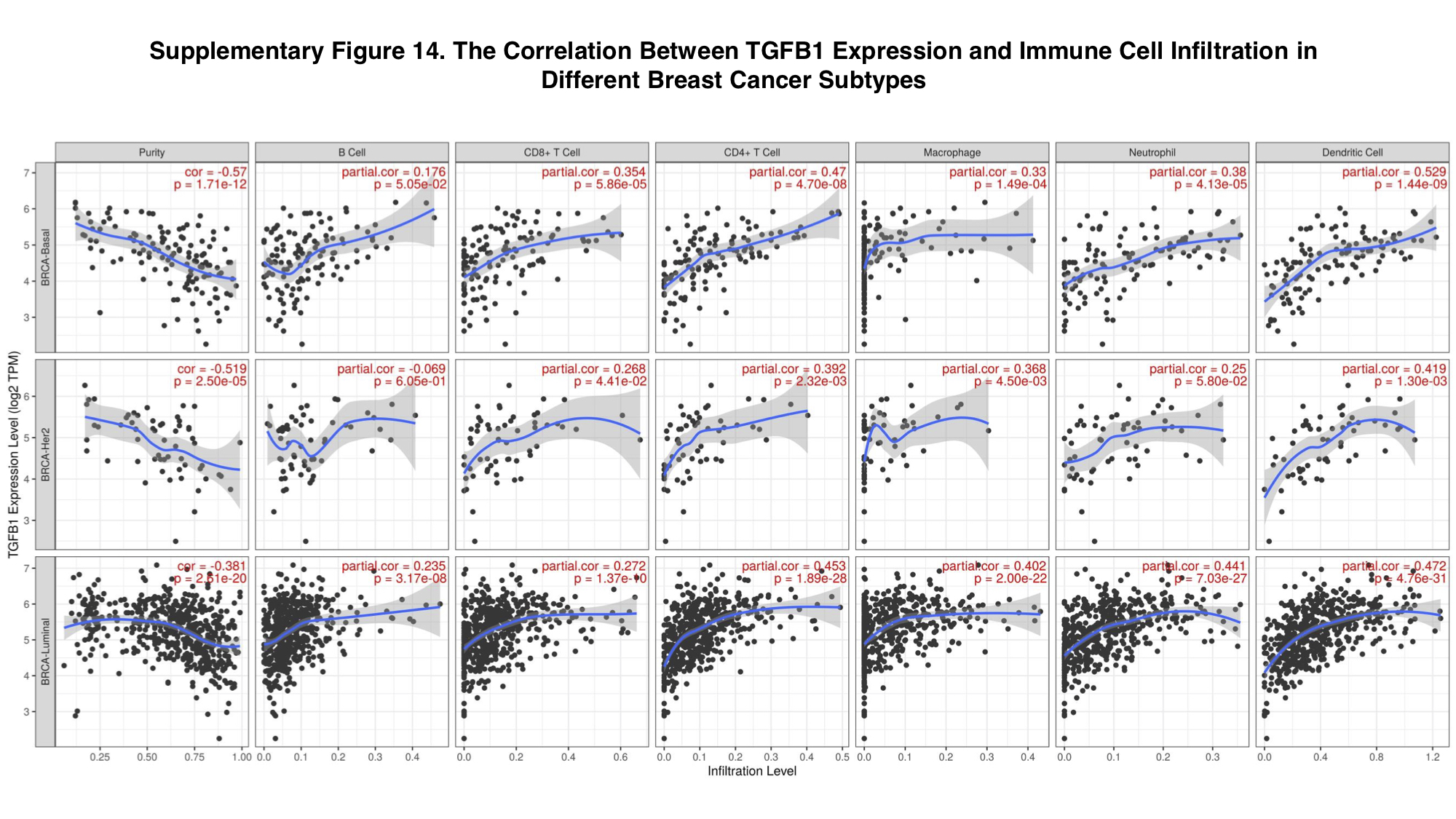

Supplement: Supplementary file 15 [file Image_14.JPEG]

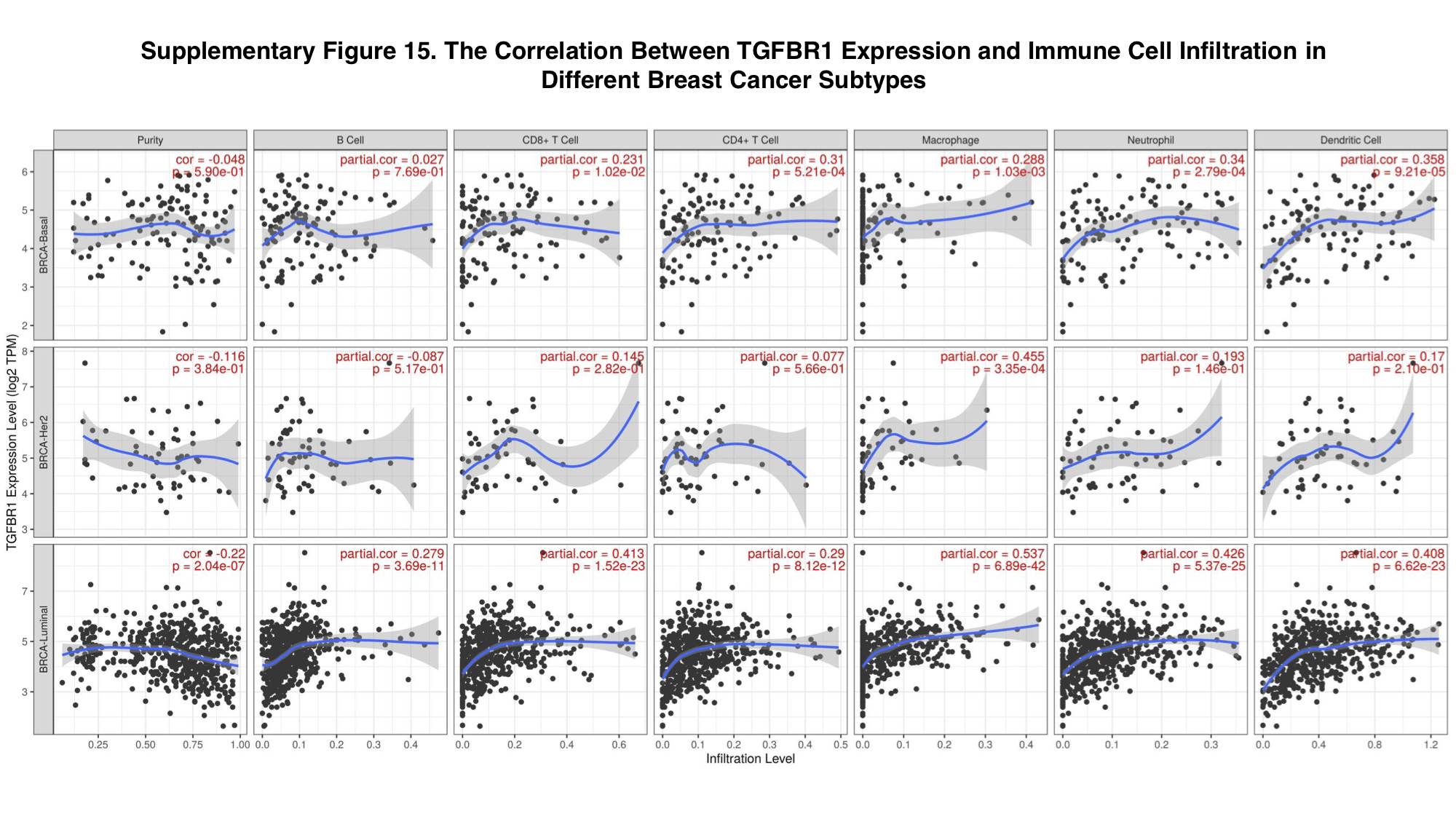

Supplement: Supplementary file 16 [file Image_15.JPEG]
