# Supplementary material for: Comprehensive Analysis of RUNX and TGF-β Mediated Regulation of Immune Cell Infiltration in Breast Cancer
Source: Front Cell Dev Biol. 2021 Aug 18;9:730380. doi: 10.3389/fcell.2021.730380 (PMC8416425; doi:10.3389/fcell.2021.730380)

Supplementary Figure 16. The Correlation Between TGFBR2 Expression and Immune Cell Infiltration in Different Breast Cancer Subtypes

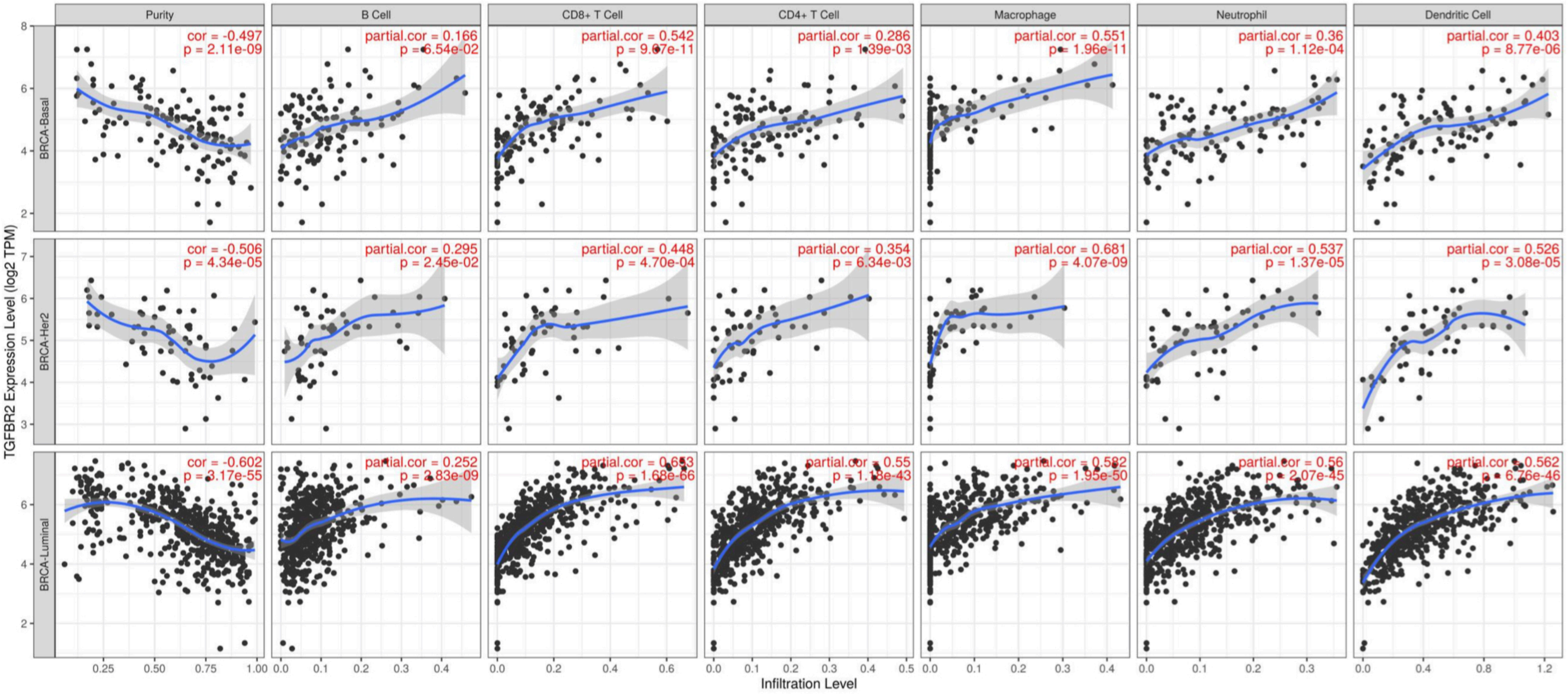

Supplement: Supplementary file 17 [file Data_Sheet_1.PDF]
