# Supplementary material for: Comprehensive Analysis of RUNX and TGF-β Mediated Regulation of Immune Cell Infiltration in Breast Cancer
Source: Front Cell Dev Biol. 2021 Aug 18;9:730380. doi: 10.3389/fcell.2021.730380 (PMC8416425; doi:10.3389/fcell.2021.730380)

Supplementary Figure 17. Correlation Between RUNX Methylation Level and TGFBR1 Expression in Breast Cancer

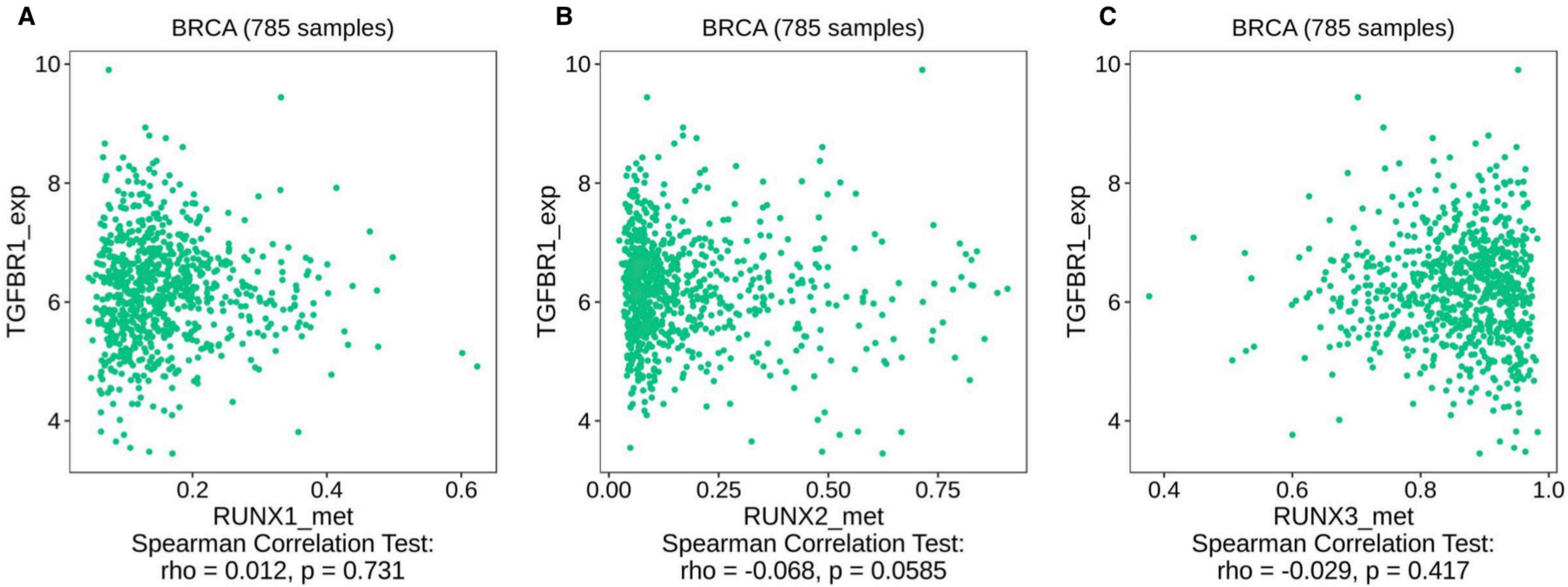

Supplement: Supplementary file 18 [file Data_Sheet_2.PDF]
